# Live Attenuated Influenza Virus as a Vector for Multivalent T-Cell Vaccines: Targeting RSV, hMPV, and PIV3

**DOI:** 10.3390/vaccines14060494

**Published:** 2026-05-30

**Authors:** Tatiana Kotomina, Pei Fong Wong, Victoria Matyushenko, Nikolay Zaramenskikh, Maria Bolgar, Anna Bazhina, Ekaterina Stepanova, Larisa Rudenko, Irina Isakova-Sivak

**Affiliations:** Department of Virology and Immunology, Institute of Experimental Medicine, 12, Acad. Pavlov Street, Saint Petersburg 197022, Russia; kotomina@iemspb.ru (T.K.); po333222@gmail.com (P.F.W.); matyshenko@iemspb.ru (V.M.); zaramenskikh.np@mail.ru (N.Z.); mariabolgar@yandex.ru (M.B.); anyta12200@gmail.com (A.B.); stepanova.ea@iemspb.ru (E.S.); vaccine@mail.ru (L.R.)

**Keywords:** live attenuated influenza vaccine (LAIV), multivalent T-cell vaccine, respiratory syncytial virus (RSV), human metapneumovirus (hMPV), parainfluenza virus type 3 (PIV3)

## Abstract

**Background/Objectives:** Respiratory syncytial virus (RSV), human metapneumovirus (hMPV), and parainfluenza virus type 3 (PIV3) are leading causes of acute respiratory infections in children and the elderly, yet no licensed T-cell vaccines are available. This study aimed to develop multivalent T-cell vaccine candidates against these pathogens using a live attenuated influenza virus (LAIV) vector platform. **Methods:** Conserved F, N, and M proteins of RSV, hMPV, and PIV3 were identified through multiple sequence alignments. Fragments enriched with experimentally confirmed and predicted T-cell epitopes were selected using the IEDB and NetMHCpan servers. These fragments were assembled into polyepitope immunogenic cassettes, and their selected order was determined by thermodynamic analysis of mRNA secondary structures using the RNAfold Web Server. The selected cassettes were cloned into the neuraminidase (NA) gene of a cold-adapted LAIV vector. Recombinant viruses were rescued by reverse genetics and assessed for replicative fitness in embryonated chicken eggs and MDCK cells, NA enzymatic activity and genetic stability upon serial passaging. **Results:** Four cassettes were designed for RSV, three for hMPV, and one for PIV3, all containing fragments with multiple T-cell epitopes. Three recombinant viruses of LAIV/RSV type and three of LAIV/hMPV type were successfully rescued, while attempts to recover the remaining recombinant viruses, i.e., LAIV/RSV and LAIV/PIV3, were not successful. All rescued recombinant viruses replicated to titers comparable to the parental LAIV strain and retained the full-length insert for at least eight passages in eggs. Importantly, NA enzymatic activity of the LAIV vector was not compromised by the insertion of the polyepitope T-cell cassettes. **Conclusions:** We developed a panel of recombinant T cell-based vaccine candidates against RSV and hMPV using the LAIV vector platform. These recombinant viruses encode conserved T-cell epitopes of the target viruses while retaining the biological properties of LAIV strains. Taken together, these characteristics warrant further evaluation of these recombinant viruses in appropriate relevant in vitro models to directly assess their immunogenicity in terms of stimulating a T-cell response against target pathogens.

## 1. Introduction

The high transmissibility of acute respiratory viral infection (ARVI) pathogens contributes significantly to their global epidemiological impact and poses a continuous threat to public health. The recent COVID-19 pandemic, caused by SARS-CoV-2, exemplified this threat, resulting in severe consequences such as a decline in global health indicators and widespread social and economic disruptions worldwide [1,2]. In addition to SARS-CoV-2, annual epidemic seasons also witness the circulation of other major pathogens like respiratory syncytial virus (RSV), human metapneumovirus (hMPV), and parainfluenza virus type 3 (PIV3), which pose a particular risk to children and can cause respiratory illnesses ranging from mild to severe, leading to significant morbidity in pediatric and elderly populations [3,4,5].

Efforts have been ongoing for many years to develop safe and effective vaccines against these pathogens. A significant milestone was reached in 2023–2024 with the approval of several vaccines for RSV prevention in older adults (aged 60 and over) in the United States and Europe, including Arexvy (GSK), Abrysvo (Pfizer), and mResvia (Moderna) [6]. Despite progress in RSV vaccine development, current technologies still face challenges in providing optimal protection for vulnerable groups like older adults and infants, necessitating alternative approaches to immunoprophylaxis [7,8]. This underscores the critical need for continued research and development focused on establishing reliable correlates of protection against respiratory diseases.

The experience with SARS-CoV-2 has underscored the importance of a coordinated adaptive immune response in effectively controlling respiratory infections. A synchronized development of early T-cell response, followed by a robust humoral response, is crucial for viral clearance and long-term protection [9]. However, respiratory viruses often evade immune surveillance, leading to recurrent infections, highlighting the need for a deeper understanding of host–pathogen interactions and host immune responses.

In this context, the development of next-generation vaccines that account for individual and population-level variations in immune responses becomes crucial. These advanced vaccines should aim to elicit broad, durable immunity, ideally at the respiratory mucosa, the primary site of pathogen entry. A promising strategy involves mimicking the immunogenicity of natural infection to induce optimal and balanced immunity [10], potentially through intranasal vaccine formulations targeting the mucosal immune system [11]. However, traditional live attenuated vaccines against RSV, hMPV, and PIV3 have faced challenges in achieving the right balance between immunogenicity and attenuation, as well as concerns about reactogenicity and genetic stability [12]. For RSV, hMPV, and PIV3, T-cell immunity plays a critical role in viral clearance and protection against reinfection [13,14,15].

As a result, a vector-based approach has emerged as a promising alternative, utilizing a safe virus as a carrier to deliver genetic material encoding immunogens from target pathogens. The live attenuated influenza vaccine (LAIV) virus, particularly the Russian donor strain A/Leningrad/134/17/57 (Len/17), has shown promise as a vector with a strong safety record and immunogenic potential in clinical trials [16,17,18].

We have extensively studied this vector as a technological platform for developing vaccines against SARS-CoV-2 in previous research [19,20,21]. In those studies, we created trivalent vaccine candidates with two chimeric type A LAIV strains (H1N1 and H3N2 subtypes). We inserted immunogenic cassettes enriched with SARS-CoV-2 T-cell epitopes into the viral neuraminidase (NA) gene segment. Immunogenicity studies in golden Syrian hamsters showed that two doses of this trivalent LAIV/CoV-2 vaccine induced both SARS-CoV-2-specific and influenza-specific T-cell immune responses, as detected by ELISPOT [22].

In our earlier work, we developed LAIV-vectored vaccines targeting RSV to stimulate T-cell immunity in a mouse model. These studies demonstrated that immunization with such vectored vaccines protected mice from viral replication in the lungs after challenge. This protection was linked to the generation of local tissue-resident memory T cells (T_RM_) in the lungs [23]. To effectively combat ARVIs, the formation of T_RM_ cells directly in the respiratory mucosa is crucial, as they offer immediate defense at the point of entry [24].

These results highlight the potential of our LAIV-based vector platform for creating T-cell vaccines against other significant respiratory pathogens like RSV, hMPV, and PIV3, which are the main focus of our current research. In this study, we outline a strategy for developing human T-cell vaccines using a modified influenza virus vector. We also describe the principles behind constructing immunogenic cassettes for stable insertion into the influenza virus genome to potentially enable the targeted induction of a broad and protective T-cell response.

## 2. Materials and Methods

### 2.1. Viruses and Plasmids

The cold-adapted live attenuated influenza vaccine (LAIV) donor strain A/Leningrad/134/17/57 (H2N2) was used as the backbone for rescuing recombinant viruses. The reverse genetics system for rescuing LAIV reassortants was established using the pCIPolISapIT vector, as previously described [25]. The hemagglutinin (HA) and neuraminidase (NA) genes were sourced from the seasonal influenza A/Guangdong-Maonan/SWL1536/2019 (H1N1) strain. The NA segment was modified to include a P2A self-cleavage site followed by a multiple cloning site for inserting immunogenic cassettes, following the strategy validated in prior studies [26]. Viral RNA of RSV, hMPV, and PIV3 was isolated from clinical isolates available at the Department of Virology and Immunology, Institute of Experimental Medicine, Saint Petersburg, Russia.

### 2.2. Selection of Conserved Target Proteins

Amino acid and nucleotide sequences of respiratory syncytial virus (RSV), human metapneumovirus (hMPV), and parainfluenza virus type 3 (PIV3) strains circulating in Europe and Asia were retrieved from the Bacterial and Viral Bioinformatics Resource Center (BV-BRC) and NCBI Protein databases. The search query included the geographical origin (Europe, Asia). For hMPV and PIV3, there were no restrictions on the year of isolation. For RSV subgroups A and B, the search was limited to the period from 2019 to 2025 to reduce data redundancy and optimize subsequent analysis steps.

Multiple sequence alignments of the amino acid sequences were conducted using the Geneious software package (version 10.2.5). Proteins with identity levels above 60% were chosen for further analysis.

### 2.3. Selection Criteria for Experimental and Predicted T-Cell Epitopes

#### 2.3.1. Experimental Epitopes

The Immune Epitope Database (IEDB) was utilized to predict potential T-cell epitopes. All experimentally confirmed human epitopes in the database were considered, regardless of the outcome of the laboratory assays used to assess the immune response. The key criterion for including an epitope in the designed immunogenic cassette was its ability to be presented by multiple human leukocyte antigen (HLA) molecules to ensure broad population coverage. The HLA alleles used for epitope prediction were those available in the NetMHCpan-4.1 and NetMHCIIpan-4.1 servers, with predicted binding affinities shown in Tables S1–S7.

#### 2.3.2. Predicted Epitopes

Potential T-cell epitopes from the N, F, and M proteins of the target viruses were selected through bioinformatic analysis using the IEDB and the NetMHCpan-4.1 (for cytotoxic T lymphocyte [CTL] epitopes) and NetMHCIIpan 4.1 (for helper T lymphocyte [HTL] epitopes) servers. The candidates underwent multi-step filtering based on criteria of immunogenicity, antigenicity (Vaxijen v.2.0, >0.4), absence of allergenicity (AllerTOP) and toxicity (ToxinPred), as well as their ability to provide coverage for the majority of circulating strains. NetMHCpan-4.1 was used with a percentile rank threshold of <0.5% for HLA class I epitopes, and NetMHCIIpan-4.1 with a threshold of <1% for HLA class II epitopes. The HLA class I panel included A1, A2, A3, A24, A26, B7, B8, B27, B39, B44, B58, and B62. For HLA class II, the panel included common HLA-DR, HLA-DQ, and HLA-DP alleles as implemented in the NetMHCIIpan-4.1 server.

### 2.4. Design of Immunogenic Cassettes

Based on the analysis discussed in the previous sections, we created polyepitope immunogenic cassettes that encode the selected antigenic fragments of the F, N, and M proteins from RSV, hMPV, and PIV3. When designing the cassettes, we considered various criteria. Each cassette’s total size was limited to 320 amino acid residues to accommodate the influenza vector’s capacity. The included fragments were chosen based on their saturation with T-cell epitopes, prioritizing regions with epitopes predicted for a wide range of HLA class I and II alleles. The design allowed for combining fragments from the same or different viral proteins within a single virus in one cassette. The selected fragments were assembled into a single polypeptide chain without flexible linker sequences to minimize the formation of unwanted neo-epitopes. Validation of the predicted epitope locations relative to the designed fragments’ boundaries was performed using multiple sequence alignment in Geneious software (version 10.2.5). Following the criteria, we selected 12 fragments (4 for each virus).

### 2.5. In Silico Thermodynamic Analysis of Cassette Variants

For each cassette, we considered several arrangements of the antigenic regions. The thermodynamic parameters of the mRNA constructs were assessed using the RNAfold Web Server [27], calculating the free energy (ΔG), enthalpy (ΔH), and entropy (ΔS) based on the nearest-neighbor model with Turner thermodynamic parameters for RNA. This analysis predicted the stability of mRNA secondary structures and the energetic favorability of their formation, guiding the selection of constructs for synthesis.

### 2.6. Construction of Recombinant NA Plasmids

We extracted viral RNA from live RSV, hMPV, and PIV3 using a standard RNA isolation kit (Biolabmix, Novosibirsk, Russia) and amplified fragments encoding the F, M, and N proteins of each virus using RT-PCR (Vazyme HiScript II One Step RT-PCR Kit, Nanjing, China). For this, specific primers were designed to include overlapping regions for subsequent gene assembly. The amplified fragments were assembled into immunogenic cassettes (RSVax-1–4, MPVax-1–3, and PIV3ax) through overlap extension PCR. These cassettes were inserted into the pCIPolISapIT vector containing the NA gene of influenza virus A/Guangdong-Maonan/SWL1536/2019 (H1N1) with a P2A self-cleavage site upstream of the insertion site. In the design, the immunogenic cassette is located downstream of P2A for co-translational cleavage from the full-length NA protein. A detailed description of the vector design can be found in our previous study [20]. Competent XL-Blue E. coli cells were transformed with the assembled plasmids, and plasmid DNA was extracted from positive clones using the Plasmid Miniprep 2.0 (Evrogen, Moscow, Russia). All constructs were verified by Sanger sequencing.

### 2.7. Virus Rescue

The recombinant influenza viruses carrying the immunogenic cassettes were generated through reverse genetics. Briefly, co-cultured HEK293T and MDCK cells were transfected with eight plasmids encoding the six internal gene segments (PB2, PB1, PA, NP, M, NS) of the Len/17 donor strain and the native HA and chimeric NA genes of the A/Guangdong-Maonan/SWL1536/2019 (H1N1) virus. The transfection was performed using GenJect-39 (Molecta, Moscow, Russia) according to the manufacturer’s instructions. At 72 h post-transfection, the supernatant was harvested and inoculated into 10-day-old embryonated chicken eggs (Sinyavino poultry farm, Leningrad Oblast, Russia) for virus amplification. The presence of the virus was confirmed by hemagglutination assay using chicken red blood cells.

### 2.8. Virus Titration in Embryonated Chicken Eggs and MDCK Cells

The infectious viral titers were determined in both embryonated chicken eggs and MDCK cells. For egg titration, serial 10-fold dilutions of the viruses were inoculated into 10-day-old eggs (0.2 mL/egg, four to five eggs per dilution) and incubated at 33 °C for 48 h. Viral growth was detected by hemagglutination assay with 0.5% chicken erythrocytes. To assess temperature sensitivity, titrations were also performed at 26 °C and 40 °C using the same protocol, with incubation for 6 days at 26 °C and 48 h at 40 °C. Determination of the titer in MDCK cell culture was performed in 96-well plates with a confluent monolayer. Serial 10-fold dilutions were prepared in DMEM medium supplemented with 1× AA and 1 μg/mL TPCK-trypsin. After adsorption, the inoculum was removed, the cells were washed, and then incubated in maintenance medium for 3 days at 33 °C and 5% CO_2_. The presence of viruses in the wells was determined by hemagglutination assay with chicken erythrocytes. The 50% infectious dose EID_50_/TCID_50_ was calculated by the Reed and Muench method [28]. The detection limits of the assays were 1.2 log_10_ EID_50_/mL and 1.8 log_10_ TCID_50_/mL.

### 2.9. Assessment of Neuraminidase Activity (ELLA)

The neuraminidase activity of recombinant viruses was assessed using the enzyme-linked lectin assay (ELLA) All viruses were amplified in 10-day-old embryonated chicken eggs and purified on a 30–60% sucrose density gradient by ultracentrifugation. Purified virus stocks were stored at –70 °C in single-use aliquots. All washing steps were performed with 120 μL per well of PBST (PBS containing 0.05% Tween-20). A 96-well high-binding plate was coated with fetuin (Sigma-Aldrich, St. Louis, MO, USA) at 50 μg/mL in carbonate-bicarbonate buffer (0.05 M, pH 9.6) at a volume of 50 μL per well overnight at 4 °C. After incubation, the plate was washed 3 times with PBST. Serial two-fold dilutions of each virus (starting from 512 HAU) were prepared in a sample diluent consisting of phosphate-buffered saline (PBS) supplemented with 1% bovine serum albumin (BSA). Then, fetuin-coated plates were incubated with 50 μL per well of virus dilutions for 1 h at 37 °C. The plate was then washed 6 times with PBST. Subsequently, 50 μL per well of PNA-HRPO (2.5 μg/mL) diluted in PBS-BSA was added. The plate was incubated for 2 h at room temperature in the dark. After 4 washes with PBST, TMB was added and the reaction was developed for 10 min, stopped with 25 μL per well of H_2_SO_4_, and OD_450_ was measured using an xMark microplate spectrophotometer (Bio-Rad, Hercules, CA, USA).

### 2.10. Assessment of Insert Genetic Stability by Serial Passaging

The genetic stability of the cassette within the NA gene was assessed by serial passaging in 10-day-old embryonated chicken eggs. The virus underwent at least eight passages, and following each passage, viral RNA was extracted from the allantoic fluid and examined using RT-PCR with primers surrounding the NA insertion site. The presence of the cassette was verified by observing the PCR product of the anticipated size on an agarose gel.

### 2.11. Statistical Analyses

Data are expressed as mean ± SD. One-way ANOVA was applied to compare viral titers among groups. Differences were considered significant at *p* < 0.05. Statistical analyses were conducted using GraphPad Prism (version 8.0).

## 3. Results

### 3.1. Assessment of Protein Conservation and Identification of Conserved Protein Candidates

Data on the amino acid sequence homology of human metapneumovirus (hMPV), respiratory syncytial virus (RSV), and parainfluenza virus type 3 (PIV3) proteins are presented in Table 1. The nucleoprotein N, matrix protein M, fusion protein F, large protein L, and matrix protein M2-1 of hMPV showed high conservation levels ranging from 83.3% to 90.3%. In contrast, the phosphoprotein P, small hydrophobic protein SH, and attachment glycoprotein G exhibited lower conservation levels and were not considered as sources of epitopes for further analysis.

Among RSV proteins, the nucleoprotein N, phosphoprotein P, matrix protein M, and protein M2-1 demonstrated high conservation levels ranging from 72.3% to 98.1%. However, the non-structural proteins NS1 and NS2, the small hydrophobic protein SH, attachment protein G, matrix protein M2-2, and large protein L showed lower conservation levels and were not suitable for inclusion in the immunogenic cassettes.

For PIV3, the nucleoprotein NP, matrix protein M, fusion protein F, phosphoprotein and hemagglutinin-neuraminidase HN exhibited high conservation levels ranging from 85.4% to 93.8%. The large protein L showed significantly lower conservation levels and was excluded from further analysis.

### 3.2. Selection of Conserved Regions Containing T-Cell Epitopes

To identify regions enriched with T-cell epitopes in the conserved F, N, and M proteins of the three viruses, we conducted an analysis of T-cell epitope distribution. We evaluated multiple experimentally confirmed (IEDB) and in silico predicted T-cell epitopes for each fragment. Fragments containing multiple epitopes were chosen as potential candidates for inclusion in immunogenic cassettes. The identified regions for respiratory syncytial virus were: RSV-N (amino acids 175–320), RSV-F (amino acids 250–320 and 404–460), and RSV-M (amino acids 180–239). The full repertoire of experimental and predicted T-cell epitopes within these regions is provided in Tables S1–S4. For human metapneumovirus, the selected fragments included MPV-N (amino acids 190–351), MPV-M (amino acids 8–69 and 190–221), and MPV-F (amino acids 6–57). The corresponding epitopes for hMPV are shown in Tables S5–S7. In the case of PIV3, no experimentally confirmed epitopes were found in the IEDB; therefore, only in silico predicted epitopes were considered. The selected fragments for PIV3 were PIV3-N (amino acids 75–115 and 318–390), PIV3-M (amino acids 300–353), and PIV3-F (amino acids 240–300). An alignment of the predicted epitopes with the parainfluenza virus type 3 protein fragments is shown in Figure S1.

### 3.3. Optimization of Fragment Arrangement Based on mRNA Structure

As a result of the mRNA secondary structure analysis, the order of fragment arrangement was selected based on the predicted mRNA stability. The selection criterion was the highest (least negative) Gibbs free energy (ΔG).

A comparison of all tested combinations led to the selection of four cassettes for RSV. The first cassette, RSVax-1, contained only the N fragment (aa 175–320) with a Gibbs free energy of –92.1 kcal/mol. The second cassette, RSVax-2, consisted of the combination M(180–239)—N(175–320) with a Gibbs free energy of −124.3 kcal/mol. The third cassette, RSVax-3, had the selected combination F(250–300)—M(180–239)—N(175–320) with a ΔG of −157.7 kcal/mol. In constructing the fourth cassette, the minimum Gibbs free energy value of −183.6 kcal/mol was achieved for two combinations: N(175–320)—F(404–460)—F(250–300)—M(180–239) and F(404–460)—M(180–239)—N(175–320)—F(250–300). The first variant, N(175–320)—F(404–460)—F(250–300)—M(180–239), was chosen for further experimentation (Table 2).

Similarly, after comparing different fragment combinations for MPV, three cassettes were chosen. The selected sequence for cassette MPVax-1 was M(190–221)—N(291–351)—M(8–69) with a ΔG of −111.1 kcal/mol. For MPVax-2, the selected combination was N(180–351)—M(190–221)—M(8–69) with a Gibbs free energy of −193.6 kcal/mol. The third cassette, MPVax-3, had the sequence F(6–57)—M(8–69)—M(190–221) and a ΔG of −97.8 kcal/mol (Table 3).

For parainfluenza virus type 3, an immunogenic cassette, PIV3ax, was designed, comprising the fragments F(240–300)—N(75–115)—N(318–390)—M(300–353) with a ΔG value of −150.9 kcal/mol (Table 4).

**Figure 1:**
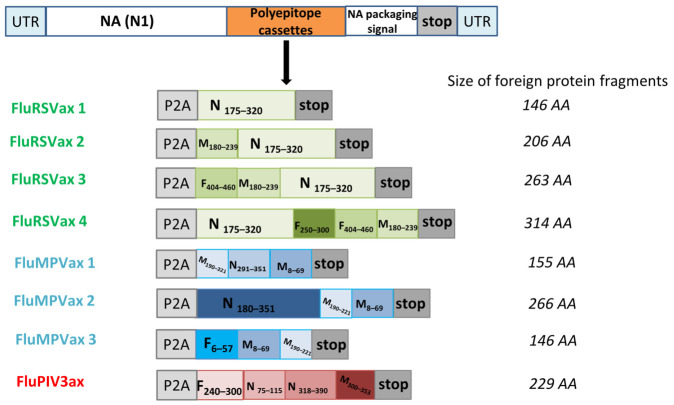
Schematic design of the recombinant LAIV NA segment carrying the immunogenic T-cell cassette. The cassette is inserted into the NA open reading frame via a P2A self-cleavage site, which facilitates independent intracellular processing of the influenza NA protein and the inserted cassette. The NA packaging signal is responsible for the incorporation of the viral RNA segment into progeny virions.

Using these selected cassettes, vaccine candidates were developed: four vaccine candidates against respiratory syncytial infection (FluRSVax), three vaccine candidates against human metapneumovirus infection (FluMPVax), and one vaccine candidate targeting parainfluenza virus type 3 (FluPIV3ax). The LAIV-NA chimeric genes and the chosen T-cell cassettes for the three target viruses are depicted in Figure 1.

Figure S2 displays the predicted secondary structures of the native influenza virus NA gene and the chimeric constructs carrying selected immunogenic cassettes for RSV (panels B–E), hMPV (panels F–H), and PIV3 (panel I). These structures show significant conformational changes introduced by the inserted sequences. Despite these alterations, all chimeric constructs maintained negative ΔG values, indicating stable RNA folding, which is a favorable feature for potential translation. The variants with the highest ΔG values were selected for virus rescue, as summarized in Table 2, Table 3 and Table 4.

### 3.4. Growth Characteristics of Recombinant Viruses in Chicken Embryos and Cell Culture

To date, we have rescued a panel of recombinant vaccine candidates, which include FluRSVax-1, FluRSVax-2, FluRSVax-3, and FluMPVax-1, FluMPVax-2, and FluMPVax-3. To determine if the insertion of immunogenic cassettes impacted viral replication ability, we compared the titers of these recombinant viruses with the parental LAIV in embryonated chicken eggs. Statistical analysis using one-way ANOVA showed no significant differences among the tested groups (*p* = 0.1353), indicating that all recombinant viruses replicated to comparable titers as the parental LAIV strain (Table 5).

To assess the impact of immunogenic cassette insertion on the cold-adapted and temperature-sensitive phenotypes, the recombinant viruses were titrated at 33 °C, 26 °C, and 40 °C in embryonated chicken eggs. Similar to the parental LAIV (titer < 1.2 log10 EID50/mL), none of the recombinant viruses replicated at 40 °C, maintaining the safety profile. However, unlike the LAIV vector, which replicated well at 26 °C (titer ~ 7.7 log_10_ EID_50_/mL), the recombinant viruses did not replicate at 26 °C (titer < 1.2 log_10_ EID_50_/mL), suggesting a potential impact of the inserted cassette on growth at low temperatures. This cold-adapted phenotype alteration has been observed in previous studies with chimeric LAIVs containing foreign inserts, although the exact mechanisms are not fully understood [26]. All recombinant viruses grew to comparable titers in MDCK cells as the parental LAIV (Table 5), indicating that the inserted cassettes did not affect viral replication capacity in cell culture. Representative titers are presented in Table 5.

### 3.5. Neuraminidase Activity of Recombinant Viruses

**Figure 2:**
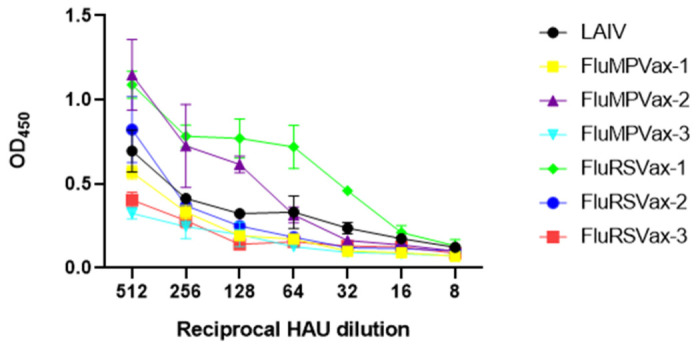
Neuraminidase activity of recombinant viruses measured by ELLA. Viruses were normalized to 512 HA units per 50 µL before the assay. Serial two-fold dilutions were incubated with fetuin-coated plates, and NA activity was detected using peanut agglutinin lectin (PNA-HRP). Data are shown as mean OD_450_ ± SD.

Neuraminidase activity was detected in all recombinant viruses, indicating that the insertion of the immunogenic cassette did not eliminate neuraminidase activity. The viruses exhibited dose-dependent NA activity, with differences in the shape of the dose–response curves observed between constructs (Figure 2). Among the constructs tested, FluRSVax-1 consistently showed the highest OD_450_ values across the dilution series, suggesting that this chimeric virus had a greater neuraminidase activity than the other vaccine candidates under the conditions tested. In contrast, the OD_450_ values for FluRSVax-2 and FluMPVax-2 were similar to those of the parental LAIV, suggesting that their neuraminidase activity was comparable to that of the parental LAIV. However, the remaining constructs (FluRSVax-3, FluMPVax-1, and FluMPVax-3) showed intermediate NA activity with a clear dose-dependent response. Overall, these results suggest that all rescued recombinant viruses exhibit detectable neuraminidase activity, albeit with varying levels among the constructs. The NA activity varied bidirectionally—some recombinants showed higher activity, while others showed a moderate decrease—indicating that the insertion of foreign T-cell cassettes did not eliminate neuraminidase function. Consistent with this, all rescued viruses showed robust replicative fitness (Table 5), as high titers require at least a basal level of functional NA.

### 3.6. Genetic Stability of Recombinant Viruses

**Figure 3:**
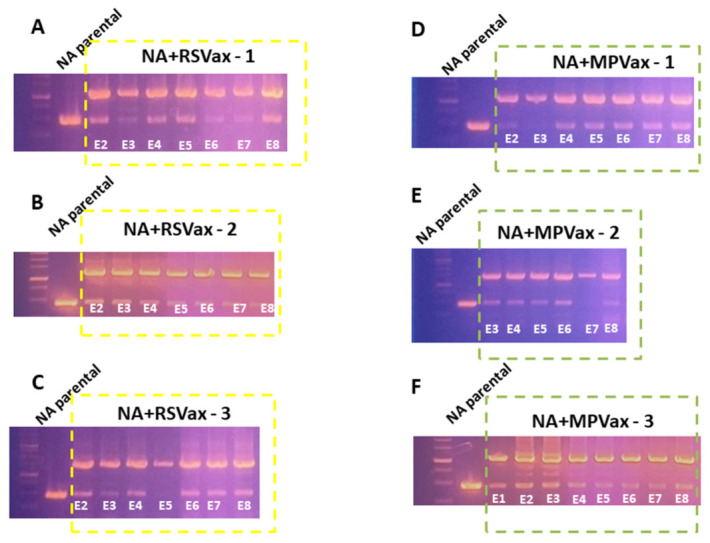
Genetic stability of recombinant LAIV vectors after serial passaging in embryonated chicken eggs. The stability of the NA gene insert in the recombinant viruses was assessed by RT-PCR for eight serial passages (E1–E8). The parental LAIV (NA parental) was used as a control in each analysis. (**A**) FluRSVax-1 (NA + RSVax1), (**B**) FluRSVax-2 (NA + RSVax2), (**C**) FluRSVax-3 (NA + RSVax3), (**D**) FluMPVax-1 (NA + MPVax-1), (**E**) FluMPVax-2 (NA + MPVax-2), and (**F**) FluMPVax-3 (NA + MPVax-3). Original gel images are shown in Figures S3–S5.

To evaluate the genetic stability of the inserts in the NA gene, the recombinant viruses were passaged eight times in embryonated chicken eggs. After each passage, viral RNA was extracted, and the NA segment was analyzed using RT-PCR with primers flanking the insertion site (Figure 3). The full-length insert was consistently present in all eight passages for the recombinant vaccine candidates FluRSVax-1, FluRSVax-2, FluRSVax-3, FluMPVax-1, FluMPVax-2, and FluMPVax-3. Sequencing of the chimeric NA genes at the eighth passage confirmed the identity of the inserts in each case. Only one amino acid substitution was detected in the FluMPVax-2 virus (Asp-33-Gly of the MPVax-2 cassette), corresponding to the N212 residue, which did not impact any of the known T-cell epitopes (Table S5). This degree of stability is similar to the typical five passages used in manufacturing processes, indicating that the NA gene can serve as a potential insertion site for immunogenic cassettes in the LAIV vector. The original gel images corresponding to the stability analysis shown in Figure 3 are provided as Figures S3–S5.

## 4. Discussion

In this study, a comparative analysis of amino acid sequences was conducted to evaluate the conservation level of proteins from RSV, hMPV, and PIV3. The fusion protein F, nucleoprotein N, and matrix protein M were identified as the most conserved proteins for inclusion in vaccine candidates. While the hemagglutinin-neuraminidase (HN) and phosphoprotein (P) of parainfluenza virus type 3 also showed high conservation, they were not included in the immunogenic cassettes due to limitations in their T-cell epitopes. HN, known for its strong humoral response [29], has predominantly conformational epitopes that may not be suitable for presentation on MHC molecules [30]. Additionally, the antibody response in children has shown that even with high titers of neutralizing antibodies, only a limited set of HN antigenic sites is recognized, with site A being the dominant one [31]. This may indicate variability in the immune response to HN within the human population. In a more recent study, Aguayo-Hiraldo et al. showed that in convalescent donors, the response to the HN protein was predominantly CD4+-mediated and variable, whereas effective cytotoxic protection requires a robust CD8+ T-cell response [15]. Similarly, the P protein, despite its conservation, was excluded from the immunogenic cassettes because it predominantly contains CD4+ T-cell epitopes and lacks CD8+ epitopes [32]. The decision to prioritize the more conserved internal N and M proteins for construct design was based on their stable linear epitopes and broader population coverage compared to HN and P proteins.

For hMPV, the large protein L, as well as the matrix proteins M2-1 and M2-2, showed high conservation in addition to the selected proteins. However, the analysis of epitope repertoire in the HMPV-TherResDB database revealed a low epitope density per unit length for the L protein, which is over 2000 amino acids in size, making it unsuitable for inclusion in compact immunogenic cassettes with a 320-amino acid limit. Due to the subdominant nature of the response to M2-2 in mice, as shown by Melendi et al., these proteins were not considered as sources of epitopes [33].

For RSV, the phosphoprotein P and the matrix protein M2-2 also exhibited high conservation in addition to the selected proteins. Our previous experiments in a mouse model showed that including the immunodominant RSV M2-1(82–90) epitope in a cassette led to the suppression of the response to the subdominant M2-1(127–135) epitope, indicating a risk of biasing the T-cell response towards a single immunodominant epitope [26]. This could potentially limit the protective potential of the vaccine candidate and the effectiveness of the immune response. Furthermore, according to data from McDermott et al., the P protein only contains CD4+ T-cell epitopes, with no CD8+ epitopes detected. Since our aim is to induce both CD4+ and CD8+ T-cell immune responses, the P protein was also excluded [32].

The M proteins of RSV and PIV3 have distinct functions, with RSV disrupting host transcription and PIV3 blocking interferon production [34,35]. The nucleoprotein N is a known T-cell target for RSV and plays a role in pathogenesis [36]. A recombinant vaccine based on the Calmette–Guérin bacillus (BCG) expressing the hRSV nucleoprotein (rBCG-hRSV-N) was developed using this target protein [37,38,39,40]. The N protein’s conservation and role in RSV pathogenesis drive interest in it as a vaccine component. Recent studies have shown an immunomodulatory function of the nucleoprotein, with high expression levels on infected cell surfaces disrupting immune synapse formation [36]. This dual interest in the N protein lies in its necessity for eliminating infected cells through T-cell responses and its accessibility as a target for monoclonal antibodies [41]. While the N protein is a well-established target for RSV vaccine candidates design, its role in hMPV and PIV3 vaccine development is less explored. The N protein has been used to create attenuated vaccine candidates for PIV3, but its immunogenic potential has not received much attention [42,43]. Attempts to modify the N gene using codon-pair deoptimization have led to excessive virus attenuation, limiting its use in vaccines [44]. Unlike RSV, where the N protein is a well-studied target for vaccinology, research on using the N protein as a target for hMPV and PIV3 vaccines is in its early stages.

For the PIV3 component, initial epitope selection was based on in silico predictions due to the lack of experimentally confirmed epitopes in IEDB at the time of analysis. However, subsequent studies have identified the Matrix (M) and Nucleoprotein (N) as dominant T-cell antigens recognized in healthy donors [15], validating the clinical relevance of T-cell immunity against PIV3 [45]. Therefore, while the initial design was predictive, it is supported by subsequent experimental evidence. The inclusion of F-derived fragments in our immunogenic cassettes aimed to broaden the T-cell response, as the fusion protein F is highly conserved across RSV, hMPV, and PIV3 and contains multiple T-cell epitopes in addition to neutralizing antibody targets.

Furthermore, the fusion protein F plays a crucial role in viral entry for RSV, hMPV, and parainfluenza virus type 3, exhibiting genetic and antigenic conservation and containing numerous neutralizing epitopes [46,47]. This makes the F protein a primary target in vaccine design against all three viruses, with current strategies focusing on stabilizing the prefusion conformation (preF) to induce a potent neutralizing antibody response [48,49,50].

In designing immunogenic cassettes to stimulate a T-cell immune response, our aim was to achieve a balanced interaction between CD4+ T helper cells and CD8+ cytotoxic T lymphocytes. This balance is crucial for effectively targeting the virus, without causing immunopathological reactions that could harm lung tissue. This is especially important for RSV, as past vaccines, such as the formalin-inactivated vaccine, have led to immune response imbalances that worsened disease upon natural infection [51]. By including CD4+ and CD8+ epitopes from conserved proteins in the construct, we aim to generate a protective immune response without causing harm.

To construct the immunogenic cassettes, fragments from different proteins of the same virus were utilized. A thermodynamic analysis of mRNA secondary structure was conducted to determine the selected order of these fragments within the cassette. mRNA stability plays a key role in translation efficiency, as overly stable structures near the start codon can impede translation initiation, while unstable transcripts are prone to degradation [52]. Variants with the highest ΔG value were selected, as this may favor translation efficiency. However, experimental validation of cassette expression is necessary, as global ΔG is only an indirect indicator of translation efficiency, with local mRNA structures near the 5’ being more critical for translation initiation.

The rescued recombinant viruses showed similar growth characteristics to the original LAIV vector in chicken eggs, suggesting that adding the immunogenic cassettes did not substantially affect viral replication. The cassettes remained genetically stable through multiple passages, confirming the NA gene as a suitable insertion site. These results indicate that our LAIV-based platform can be used for delivering foreign T-cell immunogens. The next step is to determine how to assess the immunogenicity of these constructs in vitro efficiently.

Other research groups have also utilized cold-adapted LAIV vectors to express RSV antigens. Xu et al. engineered a chimeric influenza virus using the cold-adapted A/California/7/2009 ca backbone, incorporating three repeats of the RSV F protein neutralizing epitope in the HA gene. This vaccine elicited strong RSV-specific immune responses and provided protection against RSV challenge in mice and cotton rats [53]. More recently, Deng et al. developed an NS1-deleted LAIV expressing the SARS-CoV-2 RBD, which induced neutralizing antibodies and T-cell responses and offered protection against SARS-CoV-2 challenge in mice and hamsters [54]. These studies highlight the potential of LAIV-based platforms for creating vaccines against various respiratory viruses.

A recognized limitation of LAIV-vectored vaccines is that pre-existing immunity to influenza can diminish vector immunogenicity. This is especially evident for LAIVs based on the Ann Arbor donor strain, which is approved for use only up to 18 years of age in Europe [55] and up to 49 years in the United States [56]. Various strategies have been suggested to address this limitation. One approach is heterologous prime-boost, where LAIV is followed by a different vector (e.g., adenovirus or MVA) that lacks pre-existing immunity, enabling effective boosting. Another strategy, as demonstrated by Lobby et al., involves altering immunodominant T-cell epitopes in the vector backbone. Using a mouse model, they found that pre-existing CD8+ TRM specific for a dominant influenza epitope completely blocked new T-cell responses following LAIV immunization. Notably, removing this epitope from the vector allowed sufficient evasion from cellular immunity to establish new CD8+ TRM [57]. In contrast, the Russian cold-adapted master donor virus A/Leningrad/134/17/57 (Len/17) has no age restrictions and has a proven safety and immunogenicity profile in older adults. For instance, Rudenko et al. demonstrated its efficacy and safety in nursing home residents with an average age of 74.5 years [16]. Brickley et al. showed in a clinical trial using the same Russian-backbone LAIV that higher levels of preexisting humoral and mucosal immunity correlated with reduced viral shedding and immune responses in children [58].

Direct protein-level detection of the T-cell cassette was not performed in this study due to technical challenges. T-cell epitope cassettes are designed for rapid proteasomal degradation to facilitate MHC class I presentation [59], making their detection difficult without proteasome inhibition (e.g., MG132). In our previous studies with the same LAIV vector platform and similar cassette design, we confirmed expression of inserted T-cell epitopes using functional immunological assays (ELISpot, ICS) rather than Western blot [22,60]. While direct protein-level validation is lacking, the cassette design and vector platform are consistent with our previous work, in which expression of inserted T-cell epitopes was confirmed using functional immunological assays.

All recombinant viruses in this study retained detectable neuraminidase activity despite sequence modifications in the NA gene. None of the constructs showed a complete loss of enzymatic function, indicating that a minimal level of NA activity is necessary for efficient viral release and spread. Even constructs with lower OD_450_ values (e.g., FluRSVax-2 and FluMPVax-2) reached titers comparable to the parental LAIV, suggesting that the residual NA activity was sufficient to support viral replication. These findings suggest that the NA gene can tolerate foreign insertions while retaining its detectable enzymatic activity, suggesting that the LAIV can be used as a platform for multivalent T-cell vaccine design.

Therefore, our future work will focus on establishing appropriate experimental systems to evaluate the ability of the recombinant viruses to induce specific T-cell responses in PBMCs from HLA-typed donors. A key challenge in evaluating our vaccine candidates is that they are designed to present human T-cell epitopes restricted by human HLA molecules. Standard model animals (mice, hamsters) do not express human HLA and therefore cannot be used to assess human epitope-specific T-cell responses. As discussed in our previous study [61], the limitations of HLA-transgenic mouse models for evaluating human T-cell epitopes are well recognized in the field. In our previous study, we tested whether HLA-transgenic mice expressing human HLA-A2.1 could serve as a model for immunogenicity assessment. We found that these mice are not suitable for evaluating T-cell responses to the LAIV-vectored vaccines, as immunization induced robust T-cell responses to the influenza vector but failed to elicit responses to the inserted human epitopes, due to the immunodominance of murine epitopes derived from the influenza vector itself [61]. This limitation may apply to the LAIV-vectored vaccine designed to induce human T-cell responses, as the vector backbone contains its own immunodominant murine epitopes that outcompete the inserted human epitopes in the mouse context. Therefore, we have opted for an alternative approach: in vitro immunogenicity assessment using human PBMCs from HLA-typed donors.

Despite multiple rigorous attempts, the rescue of the FluRSVax-4 and FluPIV3ax viruses has not yet yielded any viable virus. One possible explanation for this persistent difficulty lies in the individual compatibility of the chimeric NA genes encoding immunogenic cassettes, which may interfere with proper viral assembly or replication. Nevertheless, efforts to overcome this challenge continue, and we are currently exploring various modifications of transfection protocols in hopes of achieving successful virus rescue.

Other research groups have successfully used similar PBMC-based approaches to evaluate T-cell responses to vaccine candidates [62,63]. This approach allows direct evaluation of epitope presentation and T-cell activation in the relevant human context, bypassing the limitations of animal models. We recognize that the epitope selection was entirely computational, and experimental validation of epitope processing, presentation, and T-cell recognition is necessary. This is the focus of our ongoing studies using PBMCs from HLA-typed donors who have pre-existing memory T cells specific to target viruses (RSV, hMPV, PIV3). However, this technology requires further labor-intensive optimization and validation, involving blood donors with different HLA alleles. Only after a thorough and representative analysis will we be able to confidently select the most promising vaccine candidate in terms of immunogenicity from the prototypes designed and constructed in this study.

## 5. Conclusions

This study identified conserved F, N, and M proteins with the highest number of T-cell epitopes for RSV, hMPV, and PIV3. Immunogenic cassettes were designed by arranging fragments to favor the highest free energy of mRNA secondary structures. The resulting constructs (four for RSV, three for hMPV, and one for PIV3) were inserted into the neuraminidase gene of a live attenuated influenza vaccine vector. Recombinant viruses were successfully rescued for three RSV cassettes (RSVax-1, -2, -3) and all three hMPV cassettes, while RSVax-4 and PIV3ax constructs did not yield viable virus. The immunogenicity and protective efficacy of these constructs still need to be experimentally demonstrated. Future work will focus on incorporating these cassettes into H1N1 and H3N2 LAIV backbones to potentially create a trivalent seasonal influenza vaccine, providing additional T-cell immunity against RSV, hMPV, or PIV3.

## 6. Patents

No patents have resulted from the work reported in this manuscript.

## Figures and Tables

**Table 1:** Amino Acid Sequence Identity/Homology of hMPV, RSV, and PIV3 Proteins.

Target Virus	Protein	Protein Name and Type	Number of Sequences in Alignment, *n*	Mean Identity, %
hMPV	N	Nucleoprotein (structural)	188	89.1
	P	Phosphoprotein (structural)	188	67.7
	M	Matrix protein (structural)	184	90.3
	F	Fusion protein (transmembrane)	188	84.4
	M2-1	Matrix protein M2-1 (regulatory)	174	83.3
	SH	Small hydrophobic protein (transmembrane)	188	33.8
	G	Attachment glycoprotein (transmembrane)	293	60.6
	L	Large protein (polymerase/enzymatic)	188	84
RSV	NS1	Non-structural protein 1	1157	53.2
	NS2	Non-structural protein 2	1159	54
	N	Nucleoprotein (structural)	1160	98.1
	P	Phosphoprotein (structural)	1158	95.3
	M	Matrix protein (structural)	557	83.6
	SH	Small hydrophobic protein (transmembrane)	1160	29.2
	G	Attachment glycoprotein (transmembrane)	1160	17
	F	Fusion protein (transmembrane)	1163	62.2
	M2-1	Matrix protein M2-1 (regulatory)	924	72.3
	M2-2	Matrix protein M2-2 (regulatory)	923	31.3
	L	Large protein (enzymatic/polymerase)	1151	48
PIV3	N	Nucleoprotein (structural)	36	93.8
	P	Phosphoprotein (structural)	30	85.4
	M	Matrix protein (structural) (M)	44	93.8
	F	Fusion protein (transmembrane) (F)	44	89.8
	HN	Hemagglutinin-neuraminidase (HN)	43	88.6
	L	Large protein (enzymatic/polymerase) (L)	44	7.3

**Table 2:** RSV Immunogenic Cassette Combinations and Gibbs Free Energy (ΔG) Values. Cassettes were assembled from the following fragments: N(175–320), M(180–239), F1(250–300), and F2(404–460). Selected variants are highlighted in green.

Immunogenic Cassette Name	№	Combination	Gibbs Free Energy Value, kcal/mol
RSVax–1	1	N	−92.1
RSVax–2	1	N—M	−125.5
	2	M—N	−124.3
RSVax–3	1	N—M—F2	−162.8
	2	N—F2—M	−160.7
	3	M— F2—N	−162.1
	4	M—N—F2	−163.6
	5	F2—M—N	−157.7
	6	F2—N—M	−164.7
RSVax–4	1	N—M—F1—F2	−189.9
	2	N—M—F2—F1	−191.4
	3	N—F2—M—F1	−186.8
	4	N—F1—F2—M	−183.6
	5	N—F1—M—F2	−187.4
	6	N—F2—F1—M	−185.9
	7	M—N—F1—F2	−185.1
	8	M—N—F2—F1	−189.2
	9	M—F1—N—F2	−187.5
	10	M—F1—F2—N	−188.9
	11	M—F2—N—F1	−187.1
	12	M—F2—F1—N	−189.3
	13	F1—N—M—F2	−191.2
	14	F1—N—F2—M	−183.8
	15	F1—M—N—F2	−188.8
	16	F1—M—F2—N	−188.1
	17	F1—F2—N—M	−189.8
	18	F1—F2—M—N	−185.2
	19	F2—N—M—F1	−187.8
	20	F2—N—F1—M	−187.8
	21	F2—M—N—F1 *	−183.6
	22	F2—M—F1—N	−185.4
	23	F2—F1—N—M	−191.9
	24	F2—F1—M—N	−185.4

* This combination has the same ΔG value as the selected variant (highlighted in green) but was not chosen for further cloning.

**Table 3:** hMPV Immunogenic Cassette Combinations and Gibbs Free Energy (ΔG) Values. Cassettes were assembled from the following fragments: N1(291–351); N1(180–351); M1 (8–69); M2 (190–221); F(6–57). Selected variants are highlighted in green.

Immunogenic Cassette Name	№	Combination	Gibbs Free Energy Value, kcal/mol
MPVax–1	1	M1—M2—N1	−113.8
	2	M1—N1—M2	−112.9
	3	M2—N1—M1	−111.1
	4	M2—M1—N1	−112.7
	5	N1—M1—M2	−112.3
	6	N1—M2—M1	−112.1
MPVax–2	1	M1—M2—N2	−198.0
	2	M1—N2—M2	−200.0
	3	N2—M1—M2	−197.2
	4	N2—M2—M1	−193.6
	5	M2—N2—M1	−197.0
	6	M2—M1—N2	−199.8
MPVax–3	1	M1—M2—F	−102.7
	2	M1—F—M2	−101.5
	3	M2—M1—F	−102.1
	4	M2—F—M1	−101.9
	5	F—M1—M2	−97.8
	6	F—M2—M1	−99.8

**Table 4:** Parainfluenza Virus Type 3 Immunogenic Cassette Combinations and Gibbs Free Energy (ΔG) Values. Amino acid fragments of parainfluenza virus type 3 proteins used for cassette assembly: N1(75–115); N2(318–390); M(300–353); F(240–300). Selected variants are highlighted in green.

Immunogenic Cassette Name	№	Combination	Gibbs Free Energy Value, kcal/mol
PIV3ax	1	N1—N2—M—F	−153.2
	2	N1—N2—F—M	−154.6
	3	N1—M—N2—F	−155.2
	4	N1—M—F—N2	−155.0
	5	N1—F—N2—M	−159.5
	6	N1—F—M—N2	−157.2
	7	N2—N1—M—F	−154.5
	8	N2—N1—F—M	−155.2
	9	N2—M—N1—F	−160.0
	10	N2—M—F—N1	−152.4
	11	N2—F—N1—M	−154.5
	12	N2—F—M—N1	−155.1
	13	M—N1—N2—F	−157.0
	14	M—N1—F—N2	−160.5
	15	M—N2—N1—F	−156.3
	16	M—N2—F—N1	−153.7
	17	M—F—N1—N2	−153.9
	18	M—F—N2—N1	−153.4
	19	F—N1—N2—M	−150.9
	20	F—N1—M—N2	−156.3
	21	F—N2—N1—M	−152.1
	22	F—N2—M—N1	−157.8
	23	F—M—N1—N2	−157.0
	24	F—M—N2—N1	−154.5

**Table 5:** Replication of recombinant viruses at different temperatures.

Virus	Mean Viral Titers ± SD			
	Eggs, log_10_ EID_50_/mL			MDCK, log_10_ TCID_50_/mL
	33 °C	26 °C	40 °C	
LAIV	9.3 ± 0.14	7.7 ± 0.5	<1.2	5.43 ± 0.35
FluRSVax-1	8.5 ± 0.46	<1.2	<1.2	4.9 ± 0.14
FluRSVax-2	8.5 ± 0.29	<1.2	<1.2	4.55 ± 0.35
FluRSVax-3	8.6 ± 0.17	<1.2	<1.2	5.2 ± 0.85
FluMPVax-1	8.5 ± 0.3	<1.2	<1.2	5.05 ± 0.35
FluMPVax-2	8.5 ± 0.55	<1.2	<1.2	4.15 ± 0.21
FluMPVax-3	9.2 ± 0.66	<1.2	<1.2	5.45 ± 0.21

## Data Availability

The original contributions presented in this study are included in the article/Supplementary Materials. Further inquiries can be directed to the corresponding author.

## Supplementary Materials

The following supporting information can be downloaded at: https://www.mdpi.com/article/10.3390/vaccines14060494/s1, Table S1: Repertoire of experimental and predicted epitopes within the conserved, epitope-enriched region of the RSV N protein (aa 175–320). Experimental (black) and predicted (red) HTL and CTL epitopes are shown; Table S2: Repertoire of experimental and predicted epitopes within the conserved, epitope-enriched region of the RSV M protein (aa 180–239). Experimental (black) and predicted (red) HTL and CTL epitopes are shown; Table S3: Repertoire of experimental and predicted epitopes within the conserved, epitope-enriched region of the RSV F protein (aa 250–320). Experimental (black) and predicted (red) HTL and CTL epitopes are shown; Table S4: Repertoire of experimental and predicted epitopes within the conserved, epitope-enriched region of the RSV F protein (aa 404–460). Experimental (black) and predicted (red) HTL and CTL epitopes are shown; Table S5: Repertoire of experimental and predicted epitopes within the conserved, epitope-enriched region of the MPV N protein (aa 180–351). Experimental (black) and predicted (red) HTL and CTL epitopes are shown; Table S6: Repertoire of experimental and predicted epitopes within the conserved, epitope-enriched regions of the MPV M protein (aa 8–69) and MPV M protein (aa 190–221). Experimental (black) and predicted (red) HTL and CTL epitopes are shown; Table S7: Repertoire of experimental and predicted epitopes within the conserved, epitope-enriched region of the MPV F protein (aa 6–57). Experimental (black) and predicted (red) HTL and CTL epitopes are shown; Figure S1: Alignment of predicted T-cell epitopes of parainfluenza virus type 3 (PIV3) relative to the amino acid sequences of the N, M, and F proteins. Fragments selected for the construction of immunogenic cassettes are shown: (A) N (aa 75–115 and 318–390); (B) F (aa 240–300) and (C) M (aa 300–353); Figure S2: Secondary structures of native and chimeric influenza virus NA RNA variants. (A) Native NA of strain A/Guangdong-Maonan/SWL1536/2019 (H1N1). (B–E) Chimeric NA with RSV insertions: (B) NA+RSVax–1. (C) NA+RSVax–2. (D) NA+RSVax–3. (E) NA+RSVax–4. (F–H) Chimeric NA with hMPV insertions: (F) NA+MPVax–1. (G) NA+MPVax–2. (H) NA+MPVax–3. (I) Chimeric NA with parainfluenza virus type 3 insertions NA+PIV3ax. Structures are shown as Minimum Free Energy (MFE) plots generated by RNAfold; Figure S3: Original gel image of NA gene insert stability analysis (Experiment 1); Figure S4: Original gel image of NA gene insert stability analysis (Experiment 2); Figure S5: Original gel image of NA gene insert stability analysis (Experiment 3).

## Abbreviations

ANOVA	Analysis of Variance
aa	Amino acid(s)
ARVI	Acute Respiratory Viral Infection
CTL	Cytotoxic T Lymphocyte
EID_50_	50% Egg Infectious Dose
FluMPVax-1, -2, -3	Recombinant LAIV vector targeting hMPV (variants 1, 2, and 3)
FluPIV3ax	Recombinant LAIV vector targeting PIV3
FluRSVax-1, -2, -3, -4	Recombinant LAIV vector targeting RSV (variants 1, 2, 3 and 4)
HLA	Human Leukocyte Antigen
hMPV	Human Metapneumovirus
IEDB	Immune Epitope Database
LAIV	Live Attenuated Influenza Vaccine
MDCK	Madin–Darby Canine Kidney
MFE	Minimum Free Energy
MPVax	Immunogenic cassette targeting hMPV
NA	Neuraminidase
PIV3ax	Immunogenic cassette targeting PIV3
RSV	Respiratory Syncytial Virus
RSVax	Immunogenic cassette targeting RSV
RT-PCR	Reverse Transcription Polymerase Chain Reaction
T_RM_	Tissue-Resident Memory T cell
VST	Virus-Specific T cells
ΔG	Gibbs Free Energy
