# Supplementary material for: Live Attenuated Influenza Virus as a Vector for Multivalent T-Cell Vaccines: Targeting RSV, hMPV, and PIV3
Source: Vaccines (Basel). 2026 May 30;14(6):494. doi: 10.3390/vaccines14060494 (PMC13308427; doi:10.3390/vaccines14060494)
Supplement: Supplementary file 1 [file vaccines-14-00494-s001.zip › vaccines-4231104-supplementary.pdf]

Table S1. Repertoire of experimental and predicted epitopes within the conserved, epitope-enriched region of the RSV N protein (aa 175–320). Experimental (black) and predicted (red) HTL and CTL epitopes are shown.

**RSV-N (175-320)**				
Epitope ID	Epitope sequence	Location protein	CTL/HTL	Allele
10051	DRSGLTAVIRRANN	N (175-188)	—	—
—	DRSGLTAVIRRANNV	N (175-189)	HTL	HLA-DQA1*01:02; DQB1*06:02
—	SGLTAVIRR	N (177-185)	CTL	HLA-A*31:01
—	GLTAVIRRA	N(178-186)	CTL	HLA-A*02:03
21158	GLTAVIRRANNVLK	N (178-191)	—	—
—	LTAVIRRANNVLKNE	N (179-193)	HTL	HLA-DRB5*01:01; HLA-DPA1*02:01/DPB1*14:01
—	AVIRRANNV	N (181-189)	CTL	HLA-A*02:03
158700	AVIRRANNVL	N (181-190)	CTL	HLA-B*07:02
5386	AVIRRANNVLKNEM	N (181-194)	—	—
—	VIRRANNVL	N (182-190)	CTL	HLA-B*07:02
159184	VIRRANNVL	N (182-190)	CTL	HLA-B*07:02
—	IRRANNVLK	N (183-191)	CTL	HLA-A*30:01
121810	RRANNVLKNEM	N (184-194)	CTL	HLA-B*27:05; HLA-B*27:03; HLA-B*27:04; HLA-B*27:06; HLA-B*27:01; HLA-B*27:02; HLA-B*27:09
55525	RRANNVLKNEMKRY	N (184-197)	—	—
45281	NNVLKNEMKRYKGL	N (187-200)	—	—
36970	LKNEMKRYKGLLPK	N (190-203)	—	—
13385	EMKRYKGLLPKDIA	N (193-206)	—	—
121506	KRYKGLLPKDI	N (195-205)	CTL	HLA-B*27:05; HLA-B*27:03; HLA-B*27:04; HLA-B*27:06; HLA-B*27:01; HLA-B*27:02; HLA-B*27:09
56561	RYKGLLPKDIANSF	N (196-209)	—	—
21008	GLLPKDIANSFYEV	N (199-212)	—	—
158971	LPKDIANSF	N (201-209)	—	HLA-B*07:02
48041	PKDIANSFYEVFEK	N (202-215)	—	—
—	DIANSFYEV	N (204-212)	CTL	HLA-A*68:02
—	IANSFYEVF	N (205-213)	CTL	HLA-B*35:01; HLA-B*53:01; HLA-B*58:01; HLA-B*57:01
25366	IANSFYEVFEKHPH	N (205-218)		
—	NSFYEVFEK	N (207-215)	CTL	HLA-A*11:01; HLA-A*68:01
57951	SFYEVFEKHPHFID	N (208-221)	—	—
14693	EVFEKHPHFIDVFV	N (211-224)	—	—
12704	EKHPHFIDVFVHFG	N (214-227)	—	—
47814	PHFIDVFVHFGIAQ	N (217-230)	—	—
25695	IDVFVHFGIAQSST	N (220-233)	—	—
—	IDVFVHFGIAQSSTR	N (220-234)	HTL	HLA-DQA1*05:01/DQB1*03:01; HLA-DQA1*04:01/DQB1*04:02

RSV-N (175-320)				
Epitope ID	Epitope sequence	Location protein	CTL/HTL	Allele
—	DVFVHFGIAQSSTRG	—	HTL	HLA-DQA1*05:01/DQB1*03:01; HLA-DQA1*04:01/DQB1*04:02
—	VFVHFGIAQSSTRGG	—	HTL	HLA-DQA1*05:01/DQB1*03:01; HLA-DRB5*01:01
18183	FVHFGIAQSSTRGG	N (223-236)	—	—
—	FVHFGIAQSSTRGGS	—	HTL	HLA-DRB5*01:01
—	VHFGIAQSSTRGGSR	—	HTL	HLA-DRB5*01:01
15992	FGIAQSSTRGGSRV	N (226-239)	—	—
4073	AQSSTRGGSRVEGI	N (229-242)	—	—
61861	STRGGSRVEGIFAG	N (232-245)	CTL	HLA class I
20034	GGSRVEGIFAGLFM	N (235-248)	—	—
—	RVEGIFAGL	—	CTL	HLA-A*32:01
56274	RVEGIFAGLFMNAY	N (238-251)	—	—
20247	GIFAGLFMNAYGAG	N (241-254)	—	—
158750	FAGLFMNAY	N (243-251)	CTL	HLA-A*01:01
1596	AGLFMNAYGAGQVM	N (244-257)	—	—
17064	FMNAYGAGQVMLRW	N (247-260)	—	—
5783	AYGAGQVMLRWGVL	N (250-263)	CTL	HLA class I; HLA-B8
1658	AGQVMLRWGVLAKS	N (253-266)	CTL	HLA class I; HLA-A2
52746	QVMLRWGVL	N (255-263)	CTL	HLA-B8
—	QVMLRWGVL	N (255-263)	CTL	HLA-B*08:01
—	QVMLRWGVLAKSVKN	N (255-269)	HTL	HLA-DPA1*02:01/DPB1*14:01
69991	VMLRWGVLAKSVKN	N (256-269)	CTL	HLA class I
956745	VMLRWGVLAKSVKNI	N (256-270)	—	—
	MLRWGVLAK	N (257-265)	CTL	HLA-A*03:01; HLA-A*30:01
56481	RWGVLAKSVKNIML	N (259-272)	—	—
69374	VLAKSVKNIMLGHA	N (262-275)	—	—
	VLAKSVKNI	N (262-270	CTL	HLA-A*02:03
33551	KSVKNIMLGHASVQ	N (265-278)	—	—
	SVKNIMLGH	N (266-274)	CTL	HLA-A*03:01
32516	KNIMLGHASVQAEM	N (268-281)	—	—
	NIMLGHASV	N (269-277)	CTL	HLA-A*68:02
41944	MLGHASVQAEMEQV	N (271-284)	—	—
23567	HASVQAEMEQVVEV	N (274-287)	—	—
	SVQAEMEQV	N (276-284)	CTL	HLA-A*02:06; HLA-A*02:03; HLA-A*02:01; HLA-A*68:02
70524	VQAEMEQVVEVYEY	N (277-290)	—	—
	VQAEMEQVV	N (277-285)	CTL	HLA-A*02:06
	AEMEQVVEV	N (279-287)	CTL	HLA-A*02:06; HLA-B*40:01; HLA-B*44:02; HLA-B*44:03
158741	EMEQVVEVY	N (280-288)	CTL	HLA-A*01:01
	EMEQVVEVY	N (280-288)	CTL	HLA-A*01:01; HLA-A*26:01; HLA-B*35:01
13367	EMEQVVEVYEYAQK	N (280-293)	—	—

RSV-N (175-320)				
Epitope ID	Epitope sequence	Location protein	CTL/HTL	Allele
	EQVVEVYEY	N (282-290)	CTL	HLA-A*26:01; HLA-B*15:01; HLA-B*35:01; HLA-B*44:03;
	QVVEVYEYA	N (283-291)	CTL	HLA-A*02:06; HLA-A*68:02
52802	QVVEVYEYAQKLGG	N (283-296)	—	—
14912	EVYEYAQKLGGEAG	N (286-299)	—	—
14977	EYAQKLGGEAGFYH	N (289-302)	—	—
51211	QKLGGEAGFYHILN	N (292-305)	—	—
—	KLGGEAGFY	N (293-301)	CTL	HLA-A*30:02
19710	GGEAGFYHILNNPK	N (295-308)	—	—
—	GEAGFYHIL	N (296-304)	CTL	HLA-B*40:01; HLA-B*44:03; HLA-B*44:02
1516	AGFYHILNNPKASL	N (298-311)	—	HLA class I
74113	YHILNNPKASLLSL	N (301-314)	—	—
27234	ILNNPKASL	N (303-311)	—	HLA-A*02:01
38287	LNNPKASLLSLTQF	N (304-317)	—	—
45374	NPKASLLSL	N (306-314)	—	HLA-B*07:02; HLA-A*03:01; HLA-A*11:01; HLA-B*07:02
—	NPKASLLSL	N (306-314)	CTL	HLA-B*07:02; HLA-B*08:01; HLA-B*51:01; HLA-B*35:01; HLA-B*53:01
48033	PKASLLSLTQFPHF	N (307-320)	—	HLA-B*07:02
—	ASLLSLTQF	N (309-317)	CTL	HLA-A*32:01; HLA-B*58:01;HLA-B*57:01

Table S2. Repertoire of experimental and predicted epitopes within the conserved, epitope-enriched region of the RSV M protein (aa 180–239). Experimental (black) and predicted (red) HTL and CTL epitopes are shown.

RSV M (180-239)				
Epitope ID	Epitope sequence	Location protein	CTL/HTL	Allele
—	TEFKNAITNAKIIPY	M (183-197)	HTL	HLA-DRB5*01:01
920808	KNAITNAKIIPYSGL	M (186-200)	—	—
43186	NAITNAKII	M (187-195)	—	HLA-Cw4
—	NAITNAKII	M (187-195)	CTL	HLA-B*51:01
144866	AITNAKII	M (188-195)	—	HLA-B27; HLA-Cw4
158677	AITNAKIIPY	M (188-197)	—	HLA-A*01:01
1087642	NAITNAKIIPYSGLL	M (188-202)	—	HLA-DP
158869	ITNAKIIPY	M (189-197)	—	HLA-A*01:01
—	ITNAKIIPY	—	CTL	HLA-A*30:02; HLA-A*01:01; HLA-A*26:01; HLA-A*11:01; HLA-A*30:01; HLA-A*03:01; HLA-B*15:01; HLA-A*32:01

RSV M (180-239)				
Epitope	Epitope sequence	Location	CTL/HTL	Allele
1087457	AITNAKIIPYSGLL	M (189-202)	—	HLA-DP
1087458	AITNAKIIPYSGLLL	M (189-203)	—	HLA-DP
921205	NAKIIPYSGLLLVIT	M (191-205)	—	—
1087573	KIIPYSGLL	M (194-202)	—	HLA-DP
158859	IPYSGLLLVI	M (195-204)	—	HLA-B*07:02
28122	IPYSGLLLV	M (195-203)	—	HLA-B51
37900	LLVITVTDNKGAFKY	M (201-215)	—	—
1087628	LVITVTDNKGAFK	M (203-215)	—	HLA-DP
—	LVITVTDNK	—	CTL	HLA-A*11:01; HLA-A*68:01
1087775	VITVTDNKGAFK	M (204-215)	HTL	HLA-DP
—	TVTDNKGAF	—	CTL	HLA-A*26:01
159200	VTDNKGAFKY	M (206-215)	CTL	HLA-A*01:01
956752	VTDNKGAFKYIKPQS	M (206-220)	HTL	—
—	FKYIKPQSQFIVDLG	M (213-227)	HTL	HLA-DRB1*12:01
—	YIKPQSQFI	M(215-223)	CTL	HLA-A*02:03; HLA-B*08:01
956592	IKPQSQFIVDLGAYL	M (216-230)	—	—
158915	KPQSQFIVDL	M (217-226)	—	HLA-B*07:02
—	QSQFIVDLGAYLEKE	M (219-233)	HTL	HLA-DRB3*01:01; HLA-DRB3*02:02
—	FIVDLGAYL		CTL	HLA-A*02:06; HLA-A*02:01; HLA-A*02:03; HLA-A*26:01; HLA-A*68:02
956620	LGAYLEKESIYYVTT	M (226-240)	HTL	—
—	AYLEKESIY	M (228-236)	CTL	HLA-A*30:02
74635	YLEKESIYY	M (229-237)	—	HLA-A*01:01
—	LEKESIYYV	M (230-238)	CTL	HLA-B*40:01
—	SIYYVTTNW	—	—	HLA-A*26:01; HLA-A*32:01; HLA-B*57:01; HLA-B*58:01; HLA-B*53:01
—	EKESIYYVTTNWKHT	M (234-242)	HTL	HLA-DRB5*01:01
—	KESIYYVTTNWKHTA	M (232-246)	HTL	HLA-DRB5*01:01
—	ESIYYVTTNWKHTAT	M (233-247)	HTL	HLA-DPA1*01:03/DPB1*02:01
—	SIYYVTTNWKHTATR	M (234-248)	HTL	HLA-DPA1*01:03/DPB1*02:01; HLA-DPA1*01:03/DPB1*04:01

Table S3. Repertoire of experimental and predicted epitopes within the conserved, epitope-enriched region of the RSV F protein (aa 250–320). Experimental (black) and predicted (red) HTL and CTL epitopes are shown.

RSV-F (250-300)				
Epitope ID	Epitope sequence	Location protein	CTL/HTL	Allele
96647	MLTNSELLSL	F (251-260)	—	—
99471	MLTNSELLSLINDMP	F (251-265)	HTL	HLA class II
96621	LTNSELLSLI	F (252-261)	—	—
96985	TNSELLSLIN	F (253-262)	—	—
96707	NSELLSLIND	F (254-263)	—	—
96866	SELLSLINDM	F (255-264)	—	—
96246	ELLSLINDMP	F (256-265)	—	—
96583	LLSLINDMPI	F (257-266)	—	—
96615	LSLINDMPIT	F (258-268)	—	—
96894	SLINDMPITN	F (259-268)	—	—
96561	LINDMPITND	F (260-269)	—	—
96408	INDMPITNDQ	F (261-275)	—	—
99345	INDMPITNDQKKLMS	F (262-271)	HTL	HLA class II
—	INDMPITNDQKKLMS	F (261-275)	HTL	HLA-DRB1*03:01
96667	NDMPITNDQK	F (263-272)	—	—
96188	DMPITNDQKK	F (264-273)	—	—
96651	MPITNDQKKL	F (265-274)	—	—
96733	PITNDQKKLM	F (265-282)	—	—
153666	PITNDQKKLMSNNVQIVR	F (265-282)	HTL	HLA-DR3; HLA-DQ; HLA-DQ2
29005	ITNDQKKLMS	F (266-275)	—	—
96980	TNDQKKLMSN	F (267-276)	—	—
96669	NDQKKLMSNN	F (268-277)	—	—
96191	DQKKLMSNNV	F (269-278)	—	—
96777	QKKLMSNNVQ	F (270-279)	—	—
96471	KKLMSNNVQI	F (271-280)	—	—
99382	KKLMSNNVQIVRQQS	F (271-285)	HTL	HLA class II
153631	KKLMSNNVQIVRQQSYSI	F (271-288)	HTL	HLA-DQ; HLA-DR3; HLA-DQ2
96479	KLMSNNVQIV	F (272-281)	—	—
96588	LMSNNVQIVR	F (273-282)	—	—
96656	MSNNVQIVRQ	F (274-283)	—	—
96910	SNNVQIVRQQ	F (275-284)	—	—
96697	NNVQIVRQQS	F (276-285)	—	—
956658	NNVQIVRQQSYSIMS	F (276-290)	—	—
96717	NVQIVRQQSY	F (277-286)	—	—
—	NVQIVRQQSYSIMSI	F (277-291)	HTL	HLA-DRB1*15:01; HLA-DRB1*12:01
97066	VQIVRQQSYS	F (278-287)	—	—

RSV-F (250-300)				
Epitope	Epitope sequence	Location	CTL/HTL	Allele
—	VQIVRQQSY	F (278-285)	CTL	HLA-A*30:02; HLA-A*32:01; HLA-B*15:01
96772	QIVRQQSYSI	F (279-288)	—	—
96431	IVRQQSYSIM	F (280-289)	—	—
—	IVRQQSYSI	F (280-288)	CTL	HLA-A*32:01; HLA-B*08:01
97072	VRQQSYSIMS	F (281-290)	—	—
99768	VRQQSYSIMSIIKEE	F (281-295)	HTL	HLA class II
	VRQQSYSIMSIIKEE	F (281-295)	HTL	HLA-DRB1*04:05
96834	RQQSYSIMSI	F (282-291)	—	—
	RQQSYSIMSIIKEEV	F (282-296)	HTL	HLA-DRB1*04:05
96791	QQSYSIMSII	F (283-292)	—	—
96805	QSYSIMSIIK	F (284-293)	—	—
96939	SYSIMSIIKE	F (285-294)	—	—
97125	YSIMSIIKEE	F (286-295)	—	—
956769	YSIMSIIKEEVLAYV	F (286-300)	—	—
96876	SIMSIIKEEV	F (287-296)	—	—
96406	IMSIIKEEVL	F (288-297)	—	—
—	IMSIIKEEV	F (288-296)	CTL	HLA-A*02:03; HLA-A*02:01
—	MSIIKEEVL	F (289-297)	CTL	HLA-B*08:01
96655	MSIIKEEVLA	F (289-298)	—	—
96875	SIIKEEVLAY	F (290-299)	—	—
96390	IIKEEVLAYV	F (291-300)	—	—
	IIKEEVLAY	F (291-299)	CTL	HLA-A*01:01; HLA-A*30:02; HLA-A*26:01; HLA-A*32:01; HLA-B*15:01; HLA-B*35:01
	EVLAYVVQL	F (295-303)	CTL	HLA-A*02:06; HLA-A*68:02; HLA-A*26:01
	KEEVLAYVV	F (293-301)	CTL	HLA-B*40:01

Table S4. Repertoire of experimental and predicted epitopes within the conserved, epitope-enriched region of the RSV F protein (aa 404–460). Experimental (black) and predicted (red) HTL and CTL epitopes are shown.

RSV-F (404-460)				
Epitope ID	Epitope sequence	Location protein	CTL/HTL	Allele
96934	SVITSLGAIV	F (405-414)	CTL	HLA-A*02:02
97045	VITSLGAIVS	F (406-415)	CTL	HLA-A*02:02
96425	ITSLGAIVSC	F (407-416)	CTL	HLA-A*02:02
97004	TSLGAIVSCY	F (408-417)	CTL	HLA-A*02:02

RSV-F (404-460)				
Epitope	Epitope sequence	Location	CTL/HTL	Allele
96892	SLGAIVSCYG	F (409-418)	CTL	HLA-A*02:02
153709	SLGAIVSCYGKTKCTASN	F (409-426)	HTL	HLA-DRB1*15:01; HLA-DR51; HLA-DRB1*07:01
—	SLGAIVSCY	F (409-417)	CTL	HLA-A*30:02; HLA-B*15:01
96550	LGAIVSCYGK	F (410-419)	CTL	HLA-A*02:02
96308	GAIVSCYGKT	F (411-420)	CTL	HLA-A*02:02
99263	GAIVSCYGKTKCTAS	F (411-425)	HTL	HLA class II
96080	AIVSCYGKTK	F (412-421)	CTL	HLA-A*02:02
96432	IVSCYGKTKC	F (413-422)	CTL	HLA-A*02:02
97074	VSCYGKTKCT	F (414-423)	CTL	HLA-A*02:02
96858	SCYGKTKCTA	F (415-424)	CTL	HLA-A*02:02
96159	CYGKTKCTAS	F (416-425)	CTL	HLA-A*02:02
97114	YGKTKCTASN	F (417-426)	CTL	HLA-A*02:02
96323	GKTKCTASNK	F (418-427)	CTL	HLA-A*02:02
96509	KTKCTASNKN	F (419-428)	CTL	HLA-A*02:02
—	KTKCTASNK	F (419-427)	CTL	HLA-A*03:01; HLA-A*11:01; HLA-A*30:01
96965	TKCTASNKNR	F (420-429)	CTL	HLA-A*02:02
96443	KCTASNKNRG	F (421-430)	CTL	HLA-A*02:02
99366	KCTASNKNRGIIKTF	F (421-435)	HTL	HLA class II
96154	CTASNKNRGI	F (422-431)	CTL	HLA-A*02:02
96941	TASNKNRGII	F (423-432)	CTL	HLA-A*02:02
96118	ASNKNRGIIK	F (424-433)	CTL	HLA-A*02:02
96908	SNKNRGIIKT	F (425-434)	CTL	HLA-A*02:02
—	SNKNRGIIK	F (422-435)	CTL	HLA-A*30:01
96687	NKNRGIIKTF	F (426-435)	CTL	HLA-A*02:02
956655	NKNRGIIKTFSNGCD	F (426-440)	—	—
96483	KNRGIIKTFS	F (427-436)	CTL	HLA-A*02:02
153632	KNRGIIKTFSNGCDYVSN	F (427-444)	HTL	HLA-DRB1*04:01
96703	NRGIIKTFSN	F (428-437)	CTL	HLA-A*02:02
96821	RGIIKTFSNG	F (429-438)	CTL	HLA-A*02:02
96317	GIIKTFSNGC	F (430-439)	CTL	HLA-A*02:02
96391	IIKTFSNGCD	F (431-440)	CTL	HLA-A*02:02
99335	IIKTFSNGCDYVSNK	F (431-445)	HTL	HLA class II
96400	IKTFSNGCDY	F (432-441)	CTL	HLA-A*02:02
96508	KTFSNGCDYV	F (433-442)	CTL	HLA-A*02:02
96953	TFSNGCDYVS	F (434-443)	CTL	HLA-A*02:02
96302	FSNGCDYVSN	F (435-444)	CTL	HLA-A*02:02
96902	SNGCDYVSNK	F (436-445)	CTL	HLA-A*02:02
956706	SNGCDYVSNKGVDTV	F (436-450)	—	—
96673	NGCDYVSNKG	F (437-446)	CTL	HLA-A*02:02
96309	GCDYVSNKGV	F (438-447)	CTL	HLA-A*02:02
96139	CDYVSNKGVD	F (439-448)	CTL	HLA-A*02:02
96204	DYVSNKGVDT	F (440-449)	CTL	HLA-A*02:02

RSV-F (404-460)				
Epitope	Epitope sequence	Location	CTL/HTL	Allele
97133	YVSNKGVDTV	F (441-450)	CTL	HLA-A*02:02
99791	YVSNKGMDTVSVGNT	F (441-455)	HTL	HLA class II
97080	VSNKGVDTVS	F (442-451)	CTL	HLA-A*02:02
96907	SNKGVDTVSV	F (443-452)	CTL	HLA-A*02:02
96685	NKGVDTVSVG	F (444-453)	CTL	HLA-A*02:02
96458	KGVDTVSVGN	F (445-454)	CTL	HLA-A*02:02
96347	GVDTVSVGNT	F (446-455)	CTL	HLA-A*02:02
97030	VDTVSVGNTL	F (447-456)	CTL	HLA-A*02:02
96199	DTVSVGNTLY	F (448-457)	CTL	HLA-A*02:02
	DTVSVGNTL	F (448-456)	CTL	HLA-A*68:02; HLA-A*26:01
97017	TVSVGNTLYY	F (449-458)	CTL	HLA-A*02:02
	TVSVGNTLY	F (449-457)	CTL	HLA-A*01:01; HLA-A*30:02; HLA-A*26:01; HLA-B*35:01; HLA-B*15:01
97083	VSVGNTLYYV	F (450-459)	CTL	HLA-A*02:02
	VSVGNTLYY	F (450-459)	CTL	HLA-A*01:01; HLA-A*11:01; HLA-A*30:02; HLA-A*26:01; HLA-B*35:01; HLA-B*15:01; HLA-B*58:01; HLA-B*57:01
96932	SVGNTLYYVN	F (451-460)	CTL	HLA-A*02:02
	SVGNTLYYV	F (451-459)	CTL	HLA-A*02:06; HLA-A*02:01; HLA-A*02:03; HLA-A*68:02

Table S5. Repertoire of experimental and predicted epitopes within the conserved, epitope-enriched region of the MPV N protein (aa 180–351). Experimental (black) and predicted (red) HTL and CTL epitopes are shown.

MPV N (180-351)				
Epitope ID	Epitope sequence	Location protein	CTL/HTL	Allele
—	LETTVRRANRVLSDA	N (180-194)	HTL	HLA-DRB1*11:01
—	ETTVRRANR	N (181-189)	CTL	HLA-A*33:01; HLA-A*68:01
—	TTVRRANRV	N (182-190)	CTL	HLA-A*68:02
159177	TVRRANRVL	N (183-191)	CTL	HLA-B*07:02
159193	VLSDALKRY	N (190-198)	CTL	HLA-A*01:01
159244	YPRMDIPKI	N (198-206)	CTL	HLA-B*07:02
158855	IPKIARSFY	N (203-211)	CTL	HLA-B*07:02
—	IPKIARSFY	N (203-211)	CTL	HLA-B*35:01; HLA-B*53:01
—	LFEQKVYYR	N (213-221)	CTL	HLA-A*33:01; HLA-A*31:01
—	EQKVYYRSLFIEYGK	N (215-229)	HTL	HLA-DPA1*01:03/DPB1*04:01; HLA-DPA1*02:01/DPB1*01:01; HLA-DPA1*01:03/DPB1*02:01
—	QKVYYRSLFIEYGKA	N (216-230)	HTL	HLA-DPA1*01:03/DPB1*04:01; HLA-DPA1*02:01/DPB1*01:01; HLA-

MPV N (180-351)				
Epitope	Epitope sequence	Location	CTL/HTL	Allele
				DPA1*01:03/DPB1*02:01
–	KVYYRSLFI	N (217-225)	CTL	HLA-A*32:01; HLA-A*30:01
–	YYRSLFIEY	N (219-227)	CTL	HLA-A*23:01; HLA-A*24:02; HLA-A*30:02
–	RSLFIEYGK	N (221-229)	CTL	HLA-A*11:01
–	RSLFIEYGKALGSSS	N (221-235)	HTL	HLA-DQA1*05:01/DQB1*03:01
–	SLFIEYGKALGSSST	N (222-236)	HTL	HLA-DQA1*05:01/DQB1*03:01
159134	SSTGSKAESL	N (234-243)	CTL	HLA-B*07:02
	AESLFVNIF	N (240-248)	CTL	HLA-B*44:03; HLA-B*44:02; HLA-B*40:01
158789	FVNIFMQAY	N (244-252)	CTL	HLA-A*01:01
–	FVNIFMQAY	N (244-252)	CTL	HLA-A*26:01; HLA-A*01:01; HLA-A*30:02; HLA-B*35:01; HLA-B*15:01
–	QAYGAGQTM	N (250-258)	CTL	HLA-A*26:01; HLA-B*35:01; HLA-B*51:01; HLA-B*53:01; HLA-B*15:01
–	GAGQTMLRW	N (253-262)	CTL	HLA-B*58:01; HLA-B*57:01
–	QTMLRWGVIARSSNN	N (256-270)	HTL	HLA-DPA1*02:01/DPB1*14:01
–	TMLRWGVIARSSNNI	N (257-271)	HTL	HLA-DPA1*02:01/DPB1*14:01
–	MLRWGVIAR	N (258-266)	CTL	HLA-A*31:01; HLA-A*33:01
158831	IARSSNNIM	N (264-272)	CTL	HLA-B*07:02
–	SSNNIMLGH	N (267-275)	CTL	HLA-A*11:01
–	NIMLGHVSV	N (270-278)	CTL	HLA-A*02:06; HLA-A*68:02; HLA-B*08:01
–	MLGHVSVQA	N (272-280)	CTL	HLA-A*02:01; HLA-A*02:03
–	HVSVQAELK	N (275-283)	CTL	HLA-A*11:01; HLA-A*68:01
–	SVQAELKQV	N (277-285)	CTL	HLA-A*02:06; HLA-A*02:03
–	AELKQVTEV	N (280-288)	CTL	HLA-B*40:01; HLA-B*44:02; HLA-B*44:03
–	ELKQVTEVY	N (281-289)	CTL	HLA-A*26:01; HLA-A*30:02; HLA-B*15:01; HLA-B*35:01
–	KQVTEVYDL	N (283-291)	CTL	HLA-A*02:06
–	QVTEVYDLV	N (284-292)	CTL	HLA-A*68:02
–	EVYDLVREM	N (287-295)	CTL	HLA-A*26:01; HLA-A*68:02; HLA-B*35:01; HLA-B*53:01
158804	GPESGLLHL	N (296-304)	CTL	HLA-B*07:02
–	LLHLRQSPK	N (301-309)	CTL	HLA-A*03:01
–	RQSPKAGLL	N (305-313)	CTL	HLA-B*15:01
–	SPKAGLLSL	N (307-315)	CTL	HLA-B*07:02; HLA-B*08:01; HLA-B*35:01; HLA-B*51:01; HLA-B*53:01
60092	SPKAGLLSL	N (307-315)	CTL	HLA-B*07:02
6813	CPNFASVVL	N (318-326)	CTL	HLA-B*07:02
–	CPNFASVVL	N (318-326)	CTL	HLA-B*07:02; HLA-B*35:01; HLA-B*53:01; HLA-B*51:01
–	SGLGIIGMY	N (330-338)	CTL	HLA-A*30:02
–	SGLGIIGMYRGRVPN	N (330-344)	HTL	HLA-DRB1*11:01
–	GLGIIGMYRGRVPNT	N (331-345)	HTL	HLA-DRB1*11:01

**MPV N (180-351)**				
Epitope	Epitope sequence	Location	CTL/HTL	Allele
159061	RGRVPNTEL	N (339-347)	CTL	HLA-B*07:02
—	RGRVPNTEL	N (339-347)	CTL	HLA-B*07:02; HLA-A*30:01

Table S6. Repertoire of experimental and predicted epitopes within the conserved, epitope-enriched regions of the MPV M protein (aa 8–69) and MPV M protein (aa 190–221). Experimental (black) and predicted (red) HTL and CTL epitopes are shown.

MPV M (8-69)				
Epitope ID	Epitope sequence	Location protein	CTL/HTL	Allele
—	DTYQGIPYT	M (12-20)	CTL	HLA-A*68:02
—	TYQGIPYTAAVQVDL	M (13-27)	HTL	HLA-DQA1*03:01/DQB1*03:02
—	YQGIPYTAAVQVDLV	M (14-28)	HTL	HLA-DQA1*03:01/DQB1*03:02
—	QGIPYTAAVQVDLVE	M (15-29)	HTL	HLA-DRB1*09:01; HLA-DQA1*03:01/DQB1*03:02; HLA-DQA1*05:01/DQB1*02:01
—	GIPYTAAVQVDLVEK	M (16-30)	HTL	HLA-DRB1*09:01; HLA-DQA1*05:01/DQB1*02:01; HLA-DQA1*03:01/DQB1*03:02
—	IPYTAAVQV	M (12-20)	CTL	HLA-B*51:01; HLA-B*07:02; HLA-B*53:01; HLA-B*35:01
28126	IPYTAAVQV	M (12-20)	CTL	HLA-B*07:02
—	IPYTAAVQVDLVEKD	M (17-31)	HTL	HLA-DQA1*05:01/DQB1*02:01; HLA-DQA1*03:01/DQB1*03:02
—	PYTAAVQVDLVEKDL	M (18-32)	HTL	HLA-DQA1*05:01/DQB1*02:01
—	YTAAVQVDL	M (19-27)	CTL	HLA-A*68:02
—	TAAVQVDLV	M (20-28)	CTL	HLA-A*68:02
—	AVQVDLVEK	M (22-30)	CTL	HLA-A*11:01; HLA-A*03:01
—	DLLPASLTI	M (31-39)	CTL	HLA-B*51:01
38414	LPASLTIWF	M (28-36)	CTL	HLA-B*07:02
—	LPASLTIWF	M (33-41)	CTL	HLA-B*53:01; HLA-B*35:01; HLA-B*51:01; HLA-B*07:02
—	SLTIWFPLF	M (36-44)	CTL	HLA-A*23:01; HLA-A*32:01
539268	FQANTPPAV	M (39-47)	CTL	HLA-A*02:01
539268	FQANTPPAV	M (39-47)	CTL	HLA-A*02:06; HLA-A*02:03; HLA-A*02:01
—	QANTPPAVL	M (45-53)	CTL	HLA-B*07:02
159163	TPPAVLLDQL	M (43-52)	CTL	HLA-B*07:02
539268	FQANTPPAV	M (44-52)	CTL	HLA-A*02:01
159190	VLLDQLKTL	M (47-55)	CTL	HLA-A*02:01
—	KTLTITTLY	M (53-61)	CTL	HLA-A*11:01; HLA-A*01:01; HLA-A*03:01; HLA-A*30:01; HLA-A*26:01; HLA-A*30:02; HLA-A*32:01; HLA-B*57:01; HLA-B*58:01; HLA-B*15:01
158937	KTLTITTLY	M (53-61)	CTL	HLA-A*01:01
**MPV M (190-221)**				
—	LTQAKIAPYAGLIMI	M (194-208)	HTL	HLA-DRB1*15:01
—	LTQAKIAPY	M (194-202)	CTL	HLA-A*26:01; HLA-A*01:01; HLA-A*30:02; HLA-B*15:01
—	TQAKIAPYAGLIMIM	M (195-209)	HTL	HLA-DRB1*15:01
158686	ALTQAKIAPY	M (188-197)	CTL	HLA-A*01:01

158992	LTQAKIAPY	M (189-197)	CTL	HLA-A*01:01
—	TQAKIAPYA	M (195-203)	CTL	HLA-A*02:06
—	QAKIAPYAGLIMIMT	M (196-210)	HTL	HLA-DRB1*15:01
—	AKIAPYAGLIMIMTM	M (197-211)	HTL	HLA-DRB1*15:01
—	KIAPYAGLI	M (198-206)	CTL	HLA-A*32:01
25388	IAPYAGLIMI	M (194-203)	CTL	HLA class I
158691	APYAGLIMI	M (195-203)	CTL	HLA-B*07:02
	APYAGLIMI	M (195-203)	CTL	HLA-B*51:01; HLA-B*07:02; HLA-B*53:01
158692	APYAGLIMIM	M (195-204)	CTL	HLA-B*07:02
—	PYAGLIMIM	M (201-209)	CTL	HLA-A*24:02; HLA-A*23:01
159031	NPKGIFKKL	M (208-216)	CTL	HLA-B*07:02

Table S7. Repertoire of experimental and predicted epitopes within the conserved, epitope-enriched region of the MPV F protein (aa 6–57). Experimental (black) and predicted (red) HTL and CTL epitopes are shown

**MPV F (6-57)**				
Epitope ID	Epitope sequence	Location protein	CTL/HTL	Allele
—	LLITPQHGL	F (11-19)	CTL	HLA-A*02:01; HLA-A*02:03; HLA-A*02:06
158745	ESCSTITEGY	F (26-35)	CTL	HLA-A*01:01
—	SCSTITEGY	F (27-35)	CTL	HLA-A*01:01; HLA-A*30:02
159089	SCSTITEGY	F (27-35)	CTL	HLA-A*01:01
—	TITEGYLSV	F (30-38)	CTL	HLA-A*02:06; HLA-A*02:03; HLA-A*02:01; HLA-A*68:02
159230	YLSVLRTGWY	F (35-44)	CTL	HLA-A*01:01
—	YLSVLRTGW	F (35-43)	CTL	HLA-A*32:01; HLA-B*58:01; HLA-B*57:01
158990	LSVLRTGWY	F (36-44)	CTL	HLA-A*01:01
—	RTGWYTNVF	F (40-48)	CTL	HLA-A*32:01; HLA-B*58:01
—	GWYTNVFTL	F (42-50)	CTL	HLA-A*23:01; HLA-A*24:02
—	YTNVFTLEV	F (44-52)	CTL	HLA-A*02:06; HLA-A*02:01; HLA-A*02:03; HLA-A*68:02
—	LEVGDVENL	F (50-58)	CTL	HLA-B*40:01

A

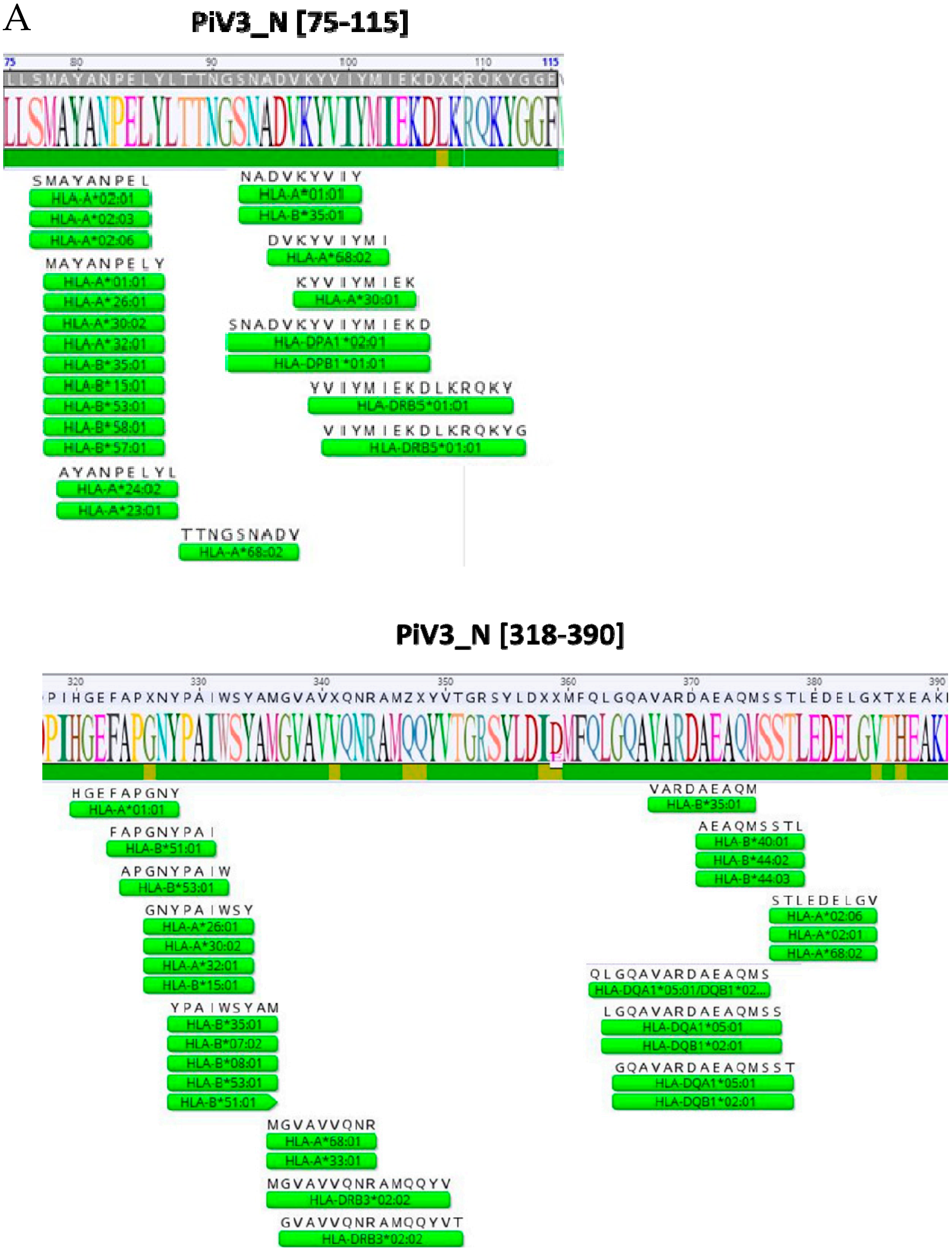

Figure S1. Alignment of predicted T-cell epitopes of parainfluenza virus type 3 (PIV3) relative to the amino acid sequences of the N, M, and F proteins. Fragments selected for the construction of immunogenic cassettes are shown: (A) N (aa 75–115 and 318–390); (B) F (aa 240–300) and (C) M (aa 300–353).

B

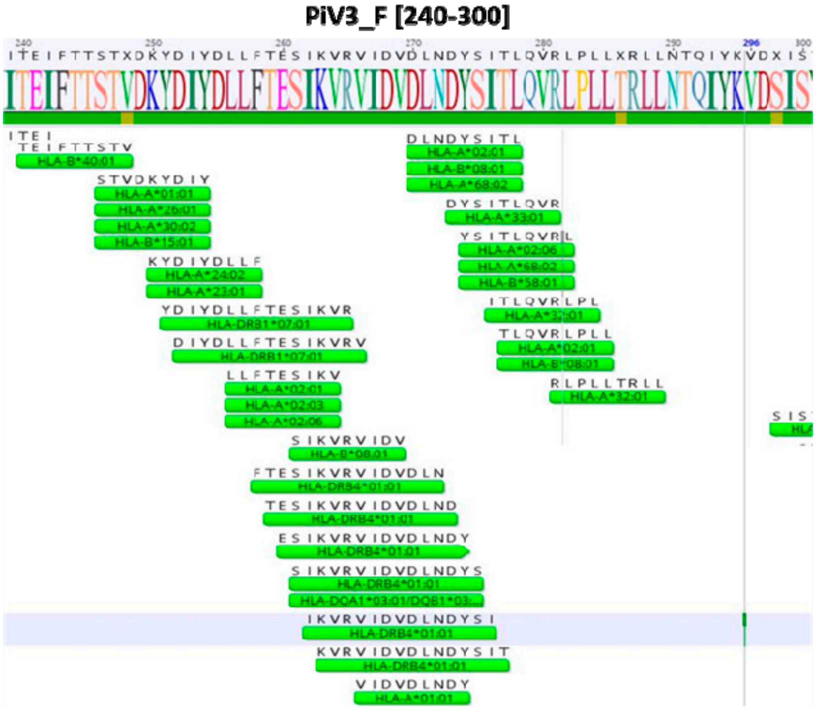

Figure S1. (continued)

C

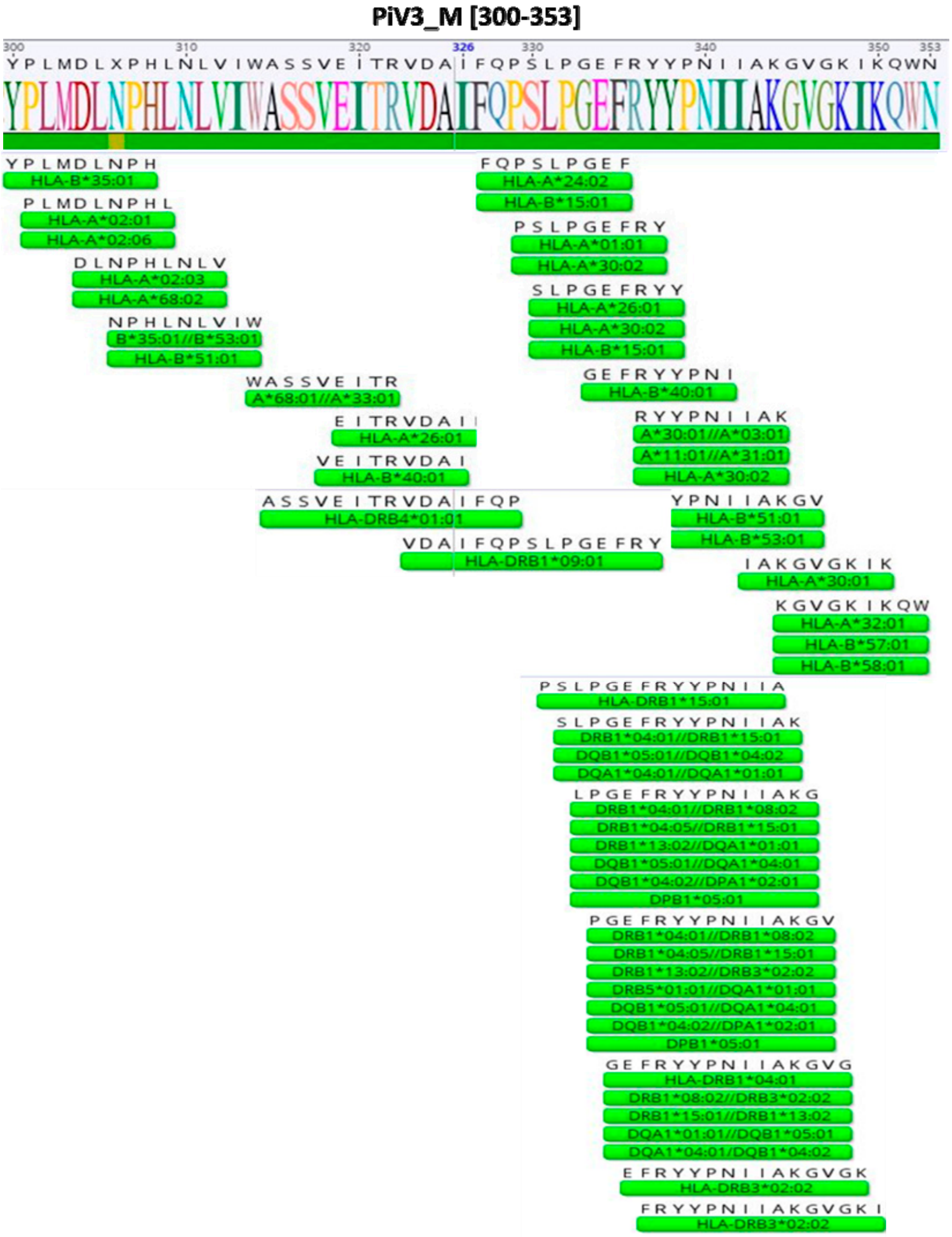

Figure S1. (continued)

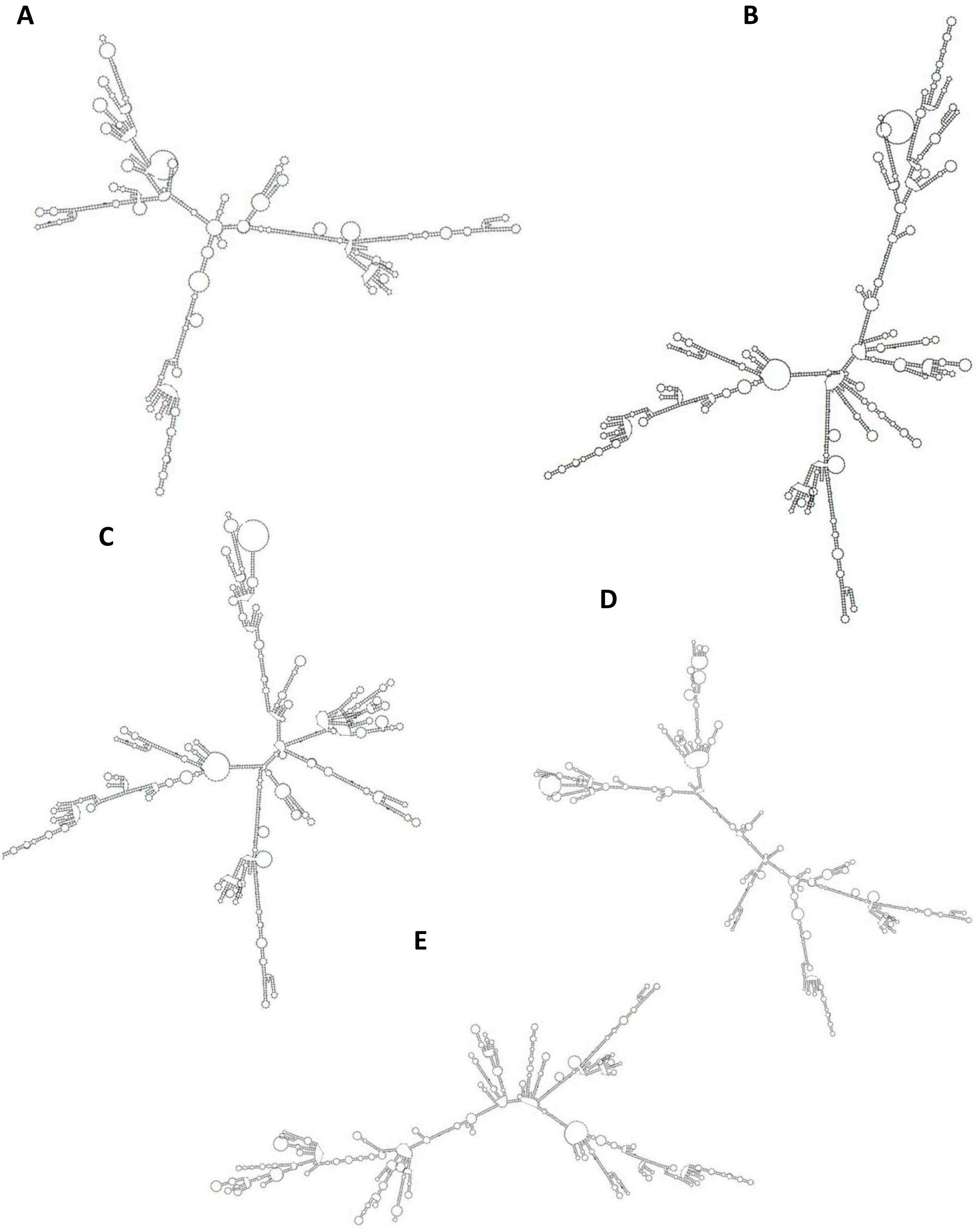

Figure S2. Secondary structures of native and chimeric influenza virus NA RNA variants. (A) Native NA of strain A/Guangdong-Maonan/SWL1536/2019 (H1N1). (B–E) Chimeric NA with RSV insertions: (B) NA+RSVax–1. (C) NA+RSVax–2. (D) NA+RSVax–3. (E) NA+RSVax–4. (F–H) Chimeric NA with hMPV insertions: (F) NA+MPVax–1. (G) NA+MPVax –2. (H) NA+MPVax –3. (I) Chimeric NA with parainfluenza virus type 3 insertions NA+ PIV3ax. Structures are shown as Minimum Free Energy (MFE) plots generated by RNAfold.

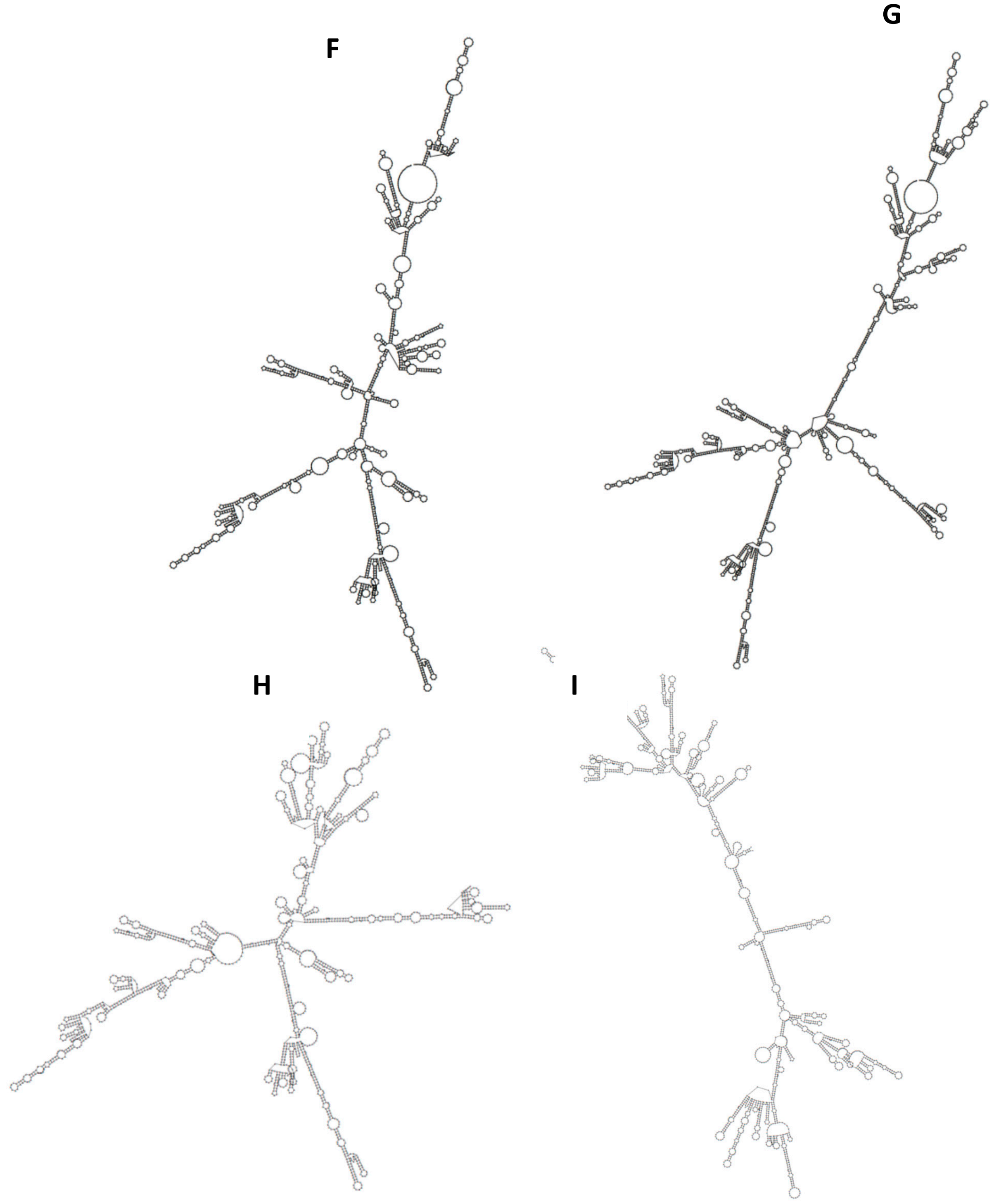

Figure S2. (Continued).

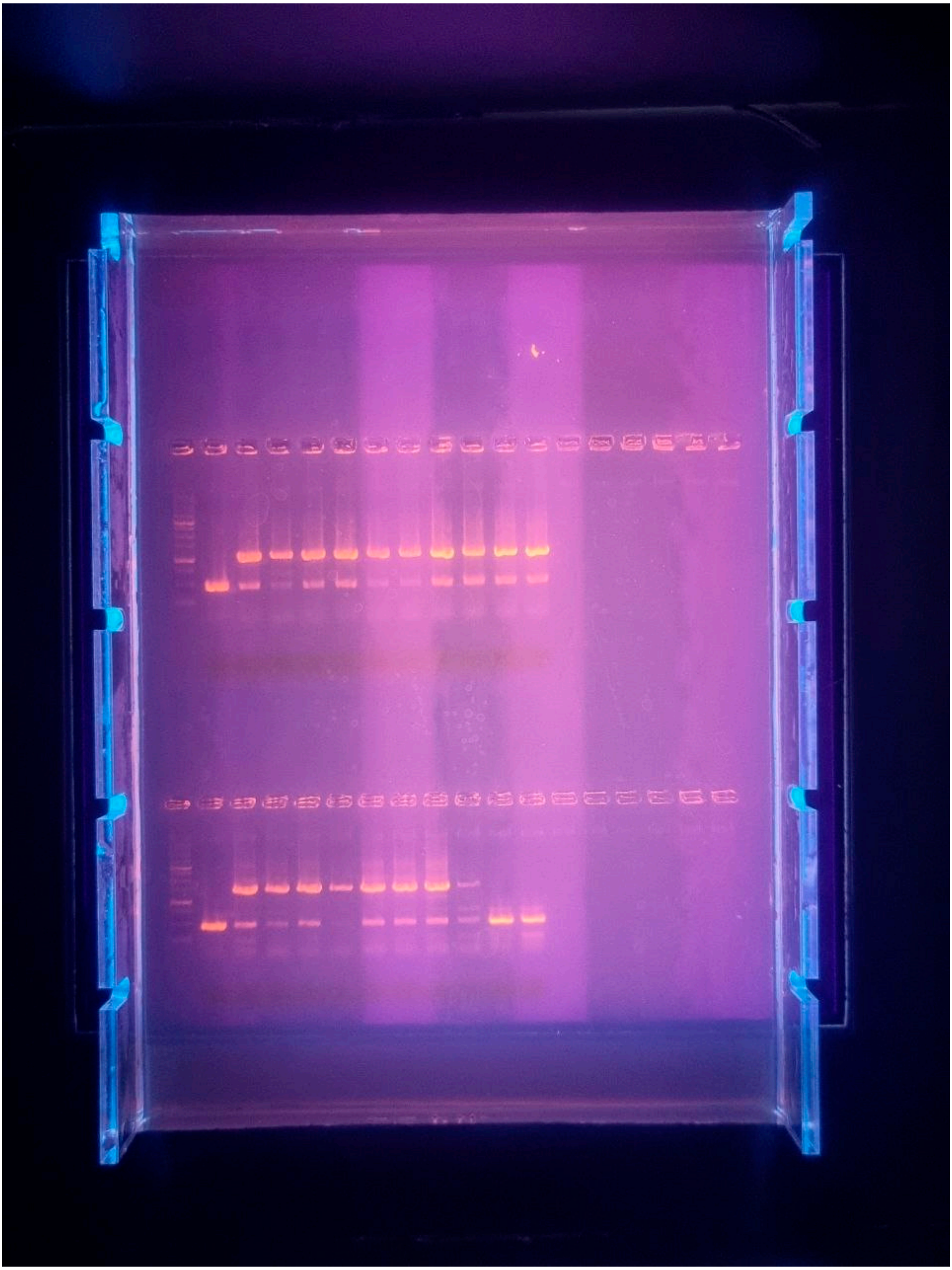

Figure S3. Original gel image of NA gene insert stability analysis (Experiment 1).

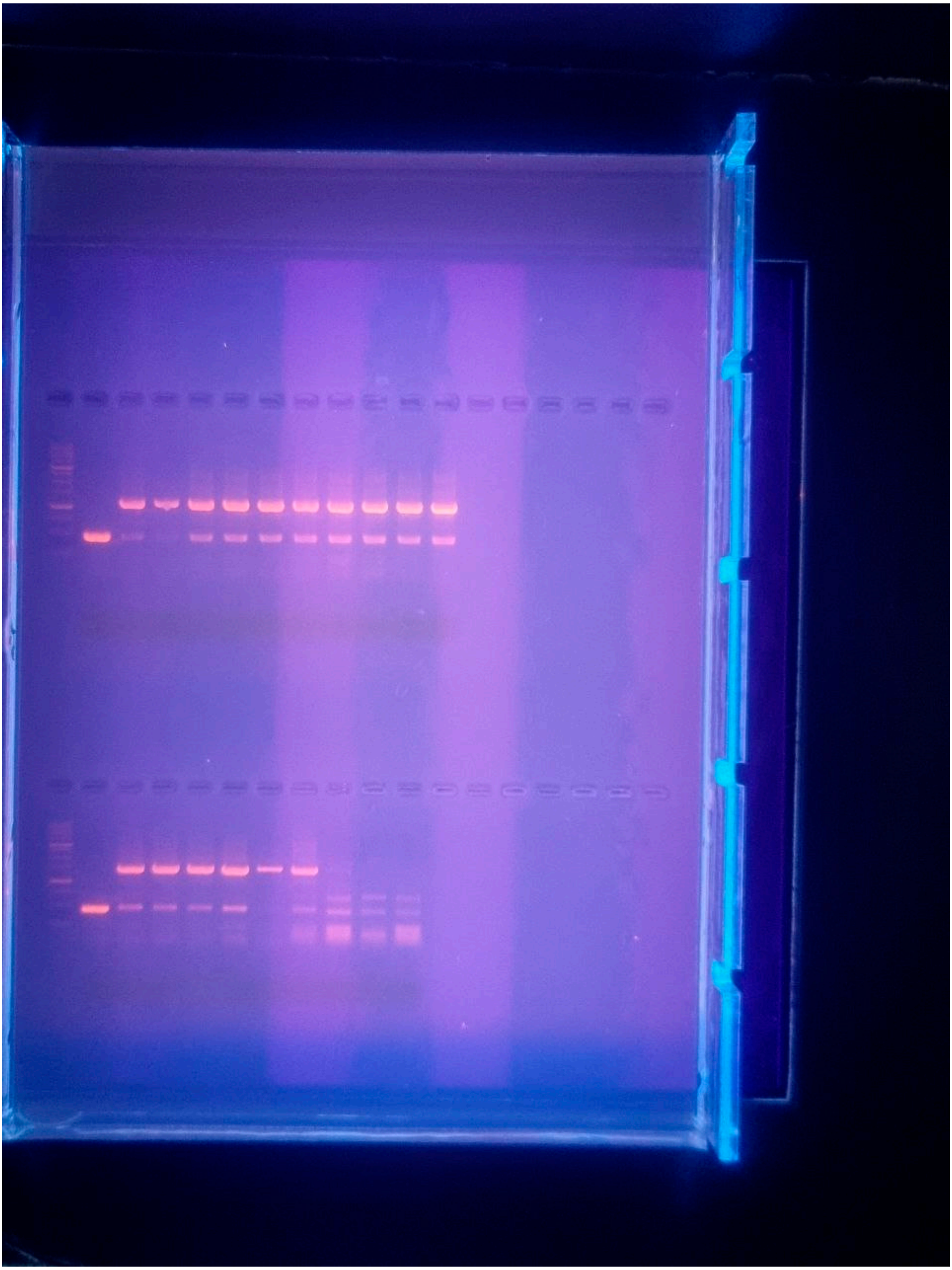

Figure S4. Original gel image of NA gene insert stability analysis (Experiment 2)

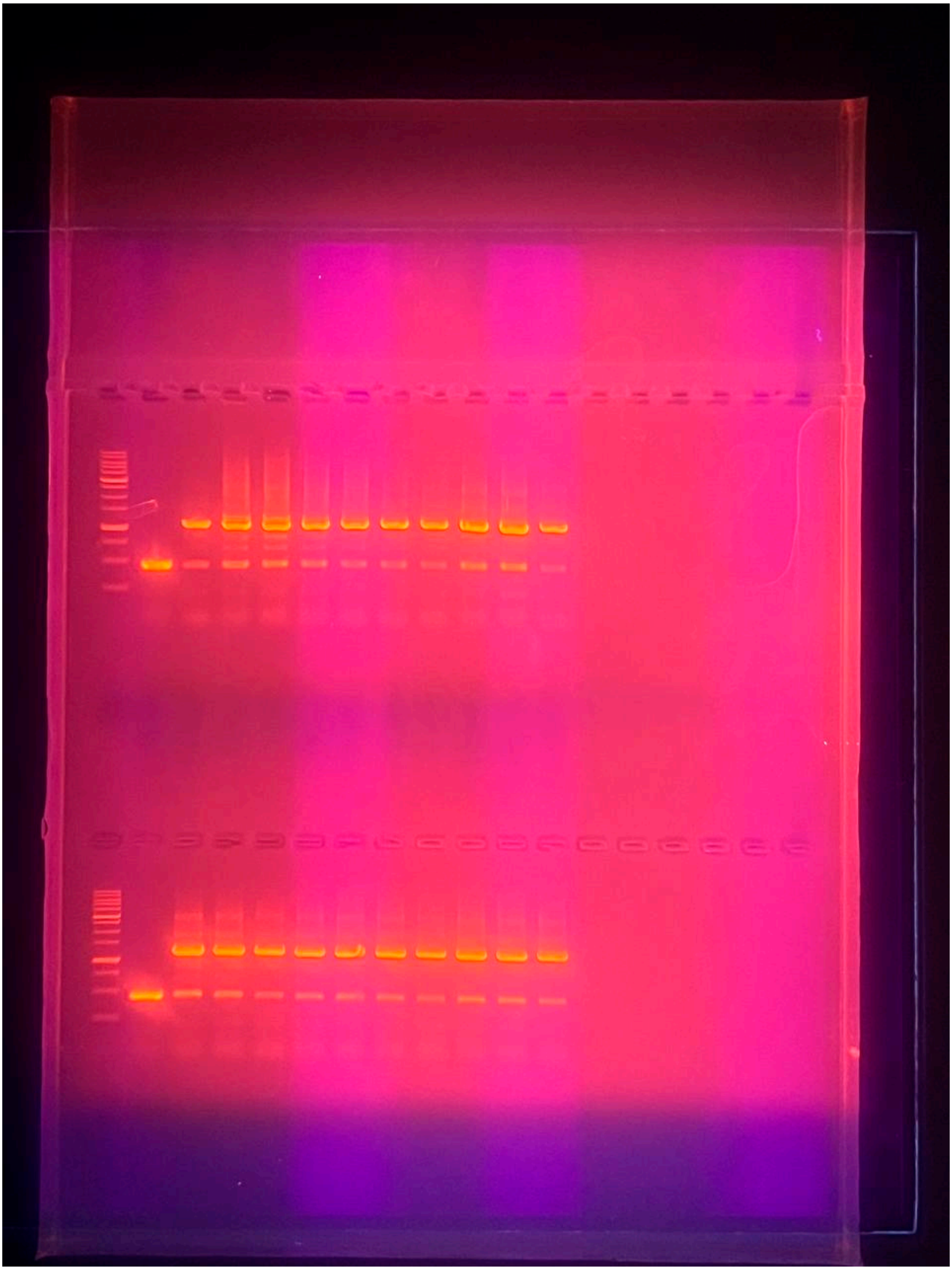

Figure S5. Original gel image of NA gene insert stability analysis (Experiment 3)
